# SIRT6 safeguards human mesenchymal stem cells from oxidative stress by coactivating NRF2

**DOI:** 10.1038/cr.2016.4

**Published:** 2016-01-15

**Authors:** Huize Pan, Di Guan, Xiaomeng Liu, Jingyi Li, Lixia Wang, Jun Wu, Junzhi Zhou, Weizhou Zhang, Ruotong Ren, Weiqi Zhang, Ying Li, Jiping Yang, Ying Hao, Tingting Yuan, Guohong Yuan, Hu Wang, Zhenyu Ju, Zhiyong Mao, Jian Li, Jing Qu, Fuchou Tang, Guang-Hui Liu

**Affiliations:** 1 National Laboratory of Biomacromolecules, Institute of Biophysics, Chinese Academy of Sciences, Beijing 100101, China; 2 Biodynamic Optical Imaging Center, College of Life Sciences, Peking University, Beijing 100871, China; 3 FSU-CAS Innovation Institute, Foshan University, Foshan, Guangdong 528000, China; 4 State Key Laboratory of Stem Cell and Reproductive Biology, Institute of Zoology, Chinese Academy of Sciences, Beijing 100101, China; 5 Gene Expression Laboratory, Salk Institute for Biological Studies, La Jolla, CA 92037, USA; 6 Department of Pathology, Carver College of Medicine, University of Iowa, Iowa City, IA 52242, USA; 7 Institute of Aging Research, Leibniz Link Partner Group on Stem Cell Aging, Hangzhou Normal University School of Medicine, Hangzhou, Zhejiang 310036, China; 8 School of life sciences and technology, Tongji University, Shanghai 200092, China; 9 The Key Laboratory of Geriatrics, Beijing Hospital & Beijing Institute of Geriatrics, Ministry of Health, Beijing 100730, China; 10 Ministry of Education Key Laboratory of Cell Proliferation and Differentiation, Beijing 100871, China; 11 Peking-Tsinghua Center for Life Sciences, Peking University, Beijing 100871, China; 12 Center for Molecular and Translational Medicine, CMTM, Beijing 100101, China; 13 Beijing Institute for Brain Disorders, Beijing 100069, China; 14 University of Chinese Academy of Sciences, Beijing 100049, China

**Keywords:** stem cell, aging, SIRT6, NRF2, oxidative stress

## Abstract

SIRT6 belongs to the mammalian homologs of Sir2 histone NAD^+^-dependent deacylase family. In rodents, SIRT6 deficiency leads to aging-associated degeneration of mesodermal tissues. It remains unknown whether human SIRT6 has a direct role in maintaining the homeostasis of mesodermal tissues. To this end, we generated *SIRT6* knockout human mesenchymal stem cells (hMSCs) by targeted gene editing. *SIRT6*-deficient hMSCs exhibited accelerated functional decay, a feature distinct from typical premature cellular senescence. Rather than compromised chromosomal stability, *SIRT6*-null hMSCs were predominately characterized by dysregulated redox metabolism and increased sensitivity to the oxidative stress. In addition, we found SIRT6 in a protein complex with both nuclear factor erythroid 2-related factor 2 (NRF2) and RNA polymerase II, which was required for the transactivation of NRF2-regulated antioxidant genes, including heme oxygenase 1 (HO-1). Overexpression of HO-1 in *SIRT6*-null hMSCs rescued premature cellular attrition. Our study uncovers a novel function of SIRT6 in maintaining hMSC homeostasis by serving as a NRF2 coactivator, which represents a new layer of regulation of oxidative stress-associated stem cell decay.

## Introduction

Stem cell exhaustion is considered as one of the hallmarks of aging^1,2^. Mesenchymal stem cells (MSCs) are adult stem cells with the potential of differentiation into mesodermal lineages, such as osteoblasts, chondrocytes, and adipocytes^3^, and play an important role in tissue regeneration^4^. Aged human and animals exhibit decreased number or diminished proliferative potential of MSCs^5,6^. Accelerated attrition of MSC pool has been observed in human stem cell and mouse models for human premature aging disorders, including Werner syndrome (WS) and Hutchinson Gilford progeria syndrome^7,8,9,10^. Transplantation of mesoderm-derived stem cells from young mice increases the lifespan and fitness of progeroid mice^11,12^. While MSC exhaustion is known as a culprit for aging-associated degeneration of mesodermal tissues^1^, the underlying genetic pathways employed by human MSCs (hMSCs) to maintain homeostasis have not been fully elucidated.

A number of intracellular and intercellular factors are known to maintain stem cell homeostasis^13,14^ and one of such factors is reactive oxygen species (ROS)^5^. ROS is normally maintained at a balanced level by cellular antioxidant systems^15^. Nuclear factor erythroid 2-related factor 2 (NRF2) is a critical redox sensor and is one of the master regulators of antioxidant responses. NRF2 binds to the antioxidant response elements (AREs) and activates the transcription of a number of antioxidant genes such as heme oxygenase 1 (HO-1) that is known for counteracting ROS^16^. The decline in NRF2-ARE activity is observed in aged cells, and may account for the oxidative stress-associated tissue degeneration^17,18^. The key factors regulating NRF2 antioxidant pathway in hMSCs, however, remain to be identified.

SIRT6 is one of the seven mammalian sirtuin homologues of yeast Sir2 longevity protein^19^. *Sirt6*-deficient mice suffered from multiple acute premature aging syndromes, which ultimately led to premature death within 1 month after birth^20^. SIRT6 contains a domain with NAD^+^-dependent deacetylase activity^21,22^, which accounts for transcriptional repression of target genes such as NF-κB, HIF1α, and c-JUN by deacetylating histone H3 lysine 9 (H3K9) at the gene promoters^23,24,25^. In addition, silencing SIRT6 in fibroblasts leads to the accelerated cellular senescence possibly due to attenuated deacetylation of H3K9 at the telomere region^26^. SIRT6 is also involved in DNA damage repair by deacetylation of H3K56 at the damaged DNA region^27,28,29^. Recently, evidence has emerged that SIRT6 protects cells from oxidative stress-associated DNA damage^21^, raising a possibility that SIRT6 could be involved in redox-related cellular homeostasis regulation.

Here we demonstrate that SIRT6 is a positive modulator of NRF2-HO-1 antioxidant pathway in hMSCs, a novel mechanism implicated in safeguarding stem cells from oxidative stress-associated functional decay.

## Results

### SIRT6-depleted hMSCs exhibit accelerated functional decay

**Figure 1:**
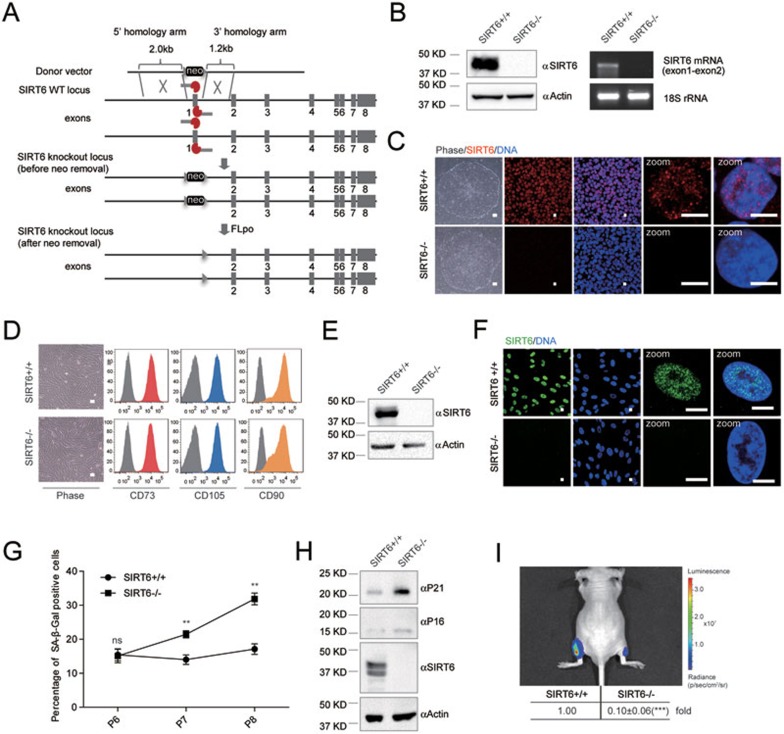
*SIRT6*-deficient hMSCs exhibit accelerated cell attrition. **(A)** Schematic representation of deletion of *SIRT6* by removing exon 1 of *SIRT6* gene via TALEN-based gene targeting technique. The donor vector contains a neomycin-resistant cassette (neo) allowing for positive selection, and the neo cassette was then removed from the *SIRT6* gene locus. **(B)** Left panel: western blotting analysis of SIRT6 protein in hESCs. Protein extracts from wild-type (WT, *SIRT6*^+/+^) and *SIRT6*-deficient (*SIRT6*^−/−^) hESCs were analyzed by western blotting using an anti-SIRT6 antibody. β-actin was used as the loading control. Right panel: RT-PCR analysis of *SIRT6* mRNA in hESCs. A pair of PCR primers spanning the junction region of *SIRT6* mRNA exon 1 and exon 2 was used. 18S rRNA was used as the loading control. **(C)** Bright-field and SIRT6 immunofluorescence micrographs of WT and *SIRT6*-deficient hESCs. DNA was stained by Hoechst 33342. Bright-field scale bar, 200 μm; immunofluorescence scale bar, 20 μm; zoom-field immunofluorescence scale bar, 10 μm. **(D)** Bright-field micrographs and FACS analysis of the surface markers CD105, CD73, and CD90 in WT and *SIRT6*-deficient hMSCs. Scale bar, 100 μm. **(E)** Western blotting analysis of SIRT6 protein in hMSCs. Protein extracts from WT and *SIRT6*-deficient hMSCs were analyzed by western blotting using anti-SIRT6 antibody. β-actin was used as the loading control. **(F)** Immunofluorescence analysis showing the absence of SIRT6 protein in the nuclei of *SIRT6*-deficient hMSCs. Scale bar, 10 μm. **(G)** SA-β-GAL staining from passage 6-8 showing an accelerated senescence in *SIRT6*-deficient hMSCs. Percentages of SA-β-GAL-positive cells were calculated. Data were presented as mean ± SEM, *n* = 5, NS, not significant, **P < 0.01. **(H)** Western blotting analysis of P16 and P21 protein in hMSCs. Protein extracts from WT and *SIRT6*-deficient hMSCs at late passage (LP, passage 9) were analyzed by western blotting. β-actin was used as the loading control. **(I)** Analysis of luciferase activity in the TA muscles of immunodeficient mice by *in vivo* imaging system (IVIS) demonstrating premature attrition of *SIRT6*-deficient hMSCs after implantation. WT (1 × 10^6^, left) and *SIRT6*-deficient (1 × 10^6^, right) hMSCs (passage 6) pretransduced with luciferase were implanted into the muscles of mice. Luciferase activities were imaged and quantified 1 week after implantation. Data were presented as mean ± SEM, *n* = 4, ***P < 0.001.

To study the role of SIRT6 in hMSCs, we first generated *SIRT6*-deficient human embryonic stem cells (hESCs). The exon 1 of *SIRT6* gene was removed in hESCs by a transcription activator-like effector nuclease (TALEN)-mediated homologous recombination strategy^30,31,32^ (Figure 1A). Biallelic loss of *SIRT6* in *SIRT6*^−/−^ hESCs was confirmed by genomic PCR and Southern blotting (Supplementary information, Figure S1A and S1B). Loss of mRNA and protein in *SIRT6*^−/−^ hESCs was verified by reverse transcription-PCR (RT-PCR) and western blotting, respectively (Figure 1B). Immunofluorescence staining demonstrated a punctate staining pattern of SIRT6 in the nuclei of wild-type (WT) hESCs, which was absent in *SIRT6*^−/−^ hESCs (Figure 1C). The *SIRT6*-deficient hESCs were cultured for more than 50 passages without discernible morphological abnormality and still exhibited defining characteristics of pluripotency, including the expression of pluripotency markers and the *in vivo* differentiation potential towards three germ layer lineages (Supplementary information, Figure S1C and S1D).

Given that tissue stem cell exhaustion has been recently recognized as one of the hallmarks of aging^10^, and the *Sirt6*-deficient mice exhibit features of premature aging in mesodermal tissues^20^, we hypothesized that *SIRT6* deficiency would result in accelerated attrition of hMSC pool. We thus differentiated the *SIRT6*^−/−^ and WT hESCs into hMSCs. *SIRT6*^−/−^ and WT hMSCs expressed typical hMSC markers such as CD73, CD90, and CD105 (Figure 1D), and were found negative for hMSC irrelevant antigens CD45, CD34 and CD43 (Supplementary information, Figure S1E)^10,31^. RT-qPCR and western blotting results confirmed the loss of both SIRT6 mRNA and protein in *SIRT6*^−/−^ hMSC (Figure 1E and Supplementary information, Figure S1F). Immunofluorescence indicated a relatively diffused localization pattern of SIRT6 in the nuclei of WT hMSCs, which was absent in the *SIRT6*^−/−^ hMSCs (Figure 1F).

As the *Sirt6*-deficient mice exhibited bone abnormalities^20^, we wondered whether the differentiation potential was compromised in *SIRT6*^−/−^ hMSCs. As expected, compared with WT hMSCs, *SIRT6*^−/−^ hMSCs exhibited substantially impaired differentiation towards osteoblasts or chondrocytes (Supplementary information, Figure S1G). Next, we investigated the link between SIRT6 and hMSC aging. Although serial passaging of *SIRT6*^−/−^ hMSCs led to several observations implicated in premature senescence such as early onset of senescence-associated (SA)-β-Gal activity (Figure 1G), upregulation of P16 and P21 proteins^33^ (Figure 1H), and progressive impairment in cell proliferation ability (Supplementary information, Figure S1H), *SIRT6*^−/−^ hMSCs also exhibited features distinct from hMSCs derived from premature aging patients^10^. For instance, different from WS-specific hMSCs^10^, decreased transcripts from centromeric repetitive sequences^34^, and a mild but significant increase of G2/M population were observed in *SIRT6*^−/−^ hMSCs (Supplementary information, Figure S1I and S1J). We ruled out the possibility that the premature aging phenotypes observed in *SIRT6*^−/−^ hMSCs resulted from chromosomal instability as reported in mouse study^20^, as *SIRT6*^−/−^ hMSCs exhibited normal karyotypes and minimal genomic mutational load determined by genome-wide copy number variation (CNV) analysis (Supplementary information, Figure S2A and S2B). Moreover, no significant increase in DNA damage response marker γH2AX was observed in *SIRT6*^−/−^ hMSCs (Supplementary information, Figure S2C and S2D). Finally, we investigated whether *SIRT6*^−/−^ hMSCs suffered from accelerated functional decay in an *in vivo* niche by transplanting WT and *SIRT6*^−/−^ hMSCs into tibialis anterior (TA) muscle of the immunodeficient mice^10^. Compared with WT hMSCs, an accelerated *in vivo* decay of *SIRT6*^−/−^ hMSCs was observed after engraftment (Figure 1I, Supplementary information, Figure S2E and S2F). The attrition of *SIRT6*^−/−^ hMSCs could be alleviated by lentiviral vector-mediated reconstitution of exogenous SIRT6 (Supplementary information, Figure S2G). Altogether, these results support that SIRT6 has a key role in preventing hMSCs from premature attrition.

### *SIRT6*^−/−^ hMSCs are susceptible to oxidative stress

**Figure 2:**
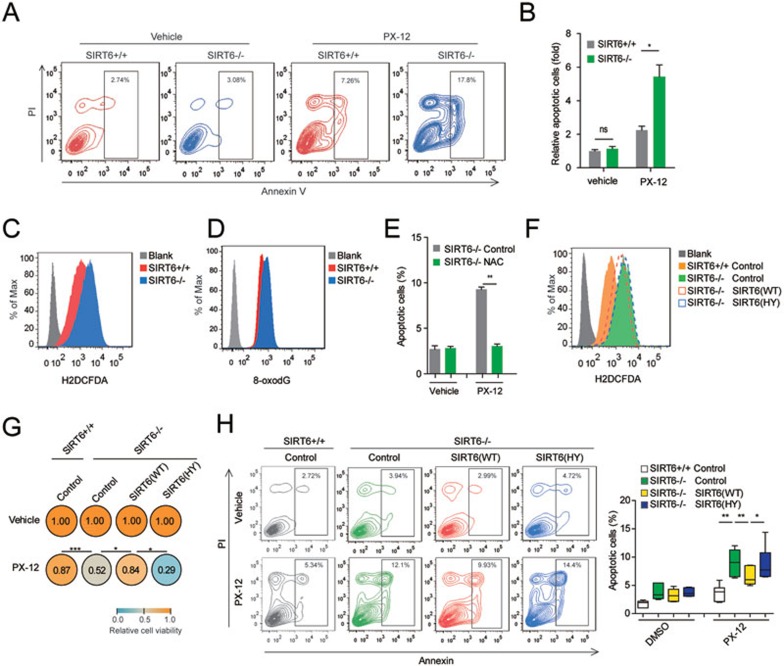
Ablation of SIRT6 in hMSCs results in elevated ROS levels and increased vulnerability to oxidative injury. **(A)** WT and *SIRT6*-deficient hMSCs were treated with vehicle (DMSO) or 50 μM PX-12 for 24 h, and the apoptotic cells were determined by Annexin V-PI staining via FACS. **(B)** Statistical analysis of **A**. Apoptotic cell percentage in vehicle-treated WT hMSCs was normalized to 1. Data were presented as mean ± SEM, *n* = 3, NS, not significant, *P < 0.05. **(C**, **D)** Cellular reactive oxygen species (ROS) and 8-oxodG levels were determined by staining with H2DCFDA probe **(C)** and an anti-8-oxodG antibody **(D)**, respectively, and measured by FACS. **(E)** *SIRT6*-deficient hMSCs were pretreated with H_2_O (control) or 1 mM NAC for 1 week, and then were treated with vehicle (DMSO) or 50 μM PX-12 for 24 h. Cellular apoptosis was measured by Annexin V-PI staining. Data were presented as mean ± SEM, *n* = 3, **P < 0.01. **(F)** Overexpression of WT SIRT6 (SIRT6 (WT)), not SIRT6 H133Y mutant (SIRT6 (HY)), in *SIRT6*-deficient hMSCs partially restored cellular ROS to normal levels. A luciferase (Control)-expressed vector was used as control. **(G**, **H)** *SIRT6*-deficient hMSCs were transduced with SIRT6 (WT), SIRT6 (HY), or Control vector, and then cells were treated with vehicle (DMSO) or 50 μM PX-12 for 24 h. Cell viability **(G)** and cellular apoptosis **(H)** were measured by MTS assay and Annexin V-PI staining, respectively. Data in **G** and **H** were presented as mean ± SEM, *n* = 6, *P < 0.05, **P < 0.01, ***P < 0.001.

To unveil how SIRT6 deficiency results in the dysregulation of hMSC homeostasis, we evaluated the sensitivity of WT and *SIRT6*^−/−^ hMSCs to various extrinsic stresses (Supplementary information, Figure S2H). PX-12, a potent inhibitor of thioredoxin and inducer of oxidative stress^35,36^, caused the strongest cytotoxicity in *SIRT6*^−/−^ hMSCs, relative to WT hMSCs (Supplementary information, Figure S2H). Treatment with 50-100 μM of PX-12, concentrations known to effectively inhibit cellular thioredoxin^37^, led to significantly reduced viability in *SIRT6*^−/−^ hMSCs by inducing apoptosis (Figure 2A, 2B and Supplementary information, Figure S2I). Similarly, *SIRT6*^−/−^ hMSCs also exhibited increased susceptibility to cytotoxicity induced by paraquat, an inducer of endogenous ROS^38,39^ (Supplementary information, Figure S2J). We observed that *SIRT6*^−/−^ hMSCs had higher basal levels of cellular ROS and DNA oxidation marker 8-oxo-7,8-dihydro-2′-deoxyguanosine (8-oxodG), relative to its WT counterparts (Figure 2C and 2D). Pretreatment of *SIRT6*^−/−^ hMSCs with the antioxidant *N*-acetylcysteine (NAC) decreased the susceptibility to PX-12-induced cytotoxicity (Figure 2E), indicating that elevated cellular ROS level is in part responsible for the increased vulnerability of *SIRT6*^−/−^ hMSCs to oxidative injury. Notably, reconstitution with WT SIRT6, but not the deacetylase-dead H133Y mutant^24,26^, not only diminished cellular ROS levels but also decreased the sensitivity of *SIRT6*^−/−^ hMSCs to PX-12 treatment (Figure 2F–2H). In addition, reintroduction of WT SIRT6, but not the H133Y mutant into *SIRT6*^−/−^ hMSCs repressed accelerated cellular senescence (Supplementary information, Figure S2K). Therefore, our data indicate that the SIRT6 deacetylase activity is crucial to counteract ROS and protect hMSCs from oxidative stress-associated functional decay.

### SIRT6 positively regulates NRF2-mediated HO-1 expression in hMSCs

**Figure 3:**
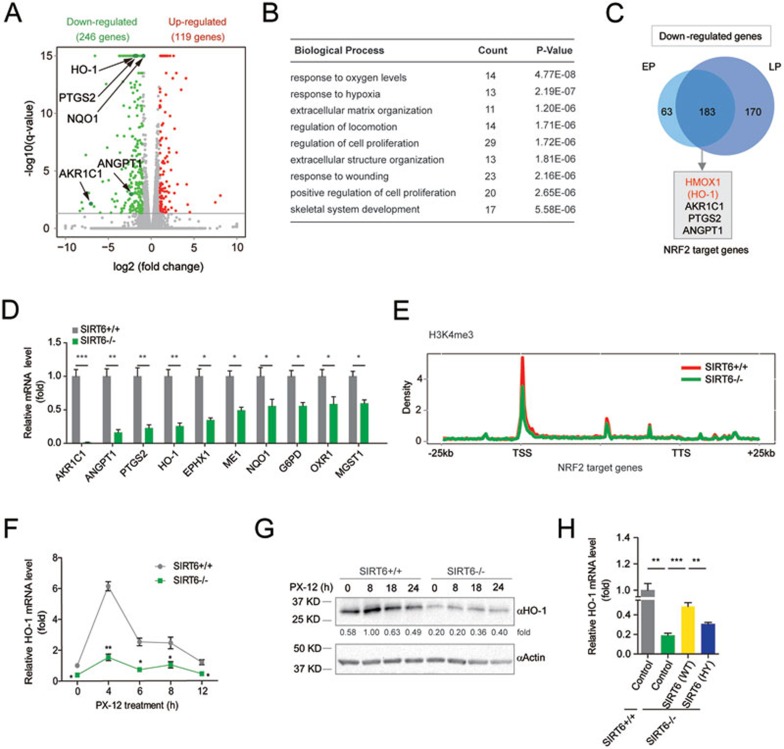
SIRT6 is required for NRF2-depedent HO-1 expression in hMSCs. **(A)** Volcano plot showing significantly altered genes (*q*-value < 0.05, FC[*SIRT6*-deficient/WT] < 0.5 or FC [*SIRT6*-deficient/WT] > 2) between WT and *SIRT6*-deficient hMSCs. Representative NRF2 target genes were highlighted (indicated by arrows). FC, fold change. **(B)** Gene ontology (GO) analysis (biological process) of significantly downregulated genes in hMSCs upon SIRT6 depletion. **(C)** Venn diagram showing that early passage (EP, passage 6) and late passage (LP, passage 9) hMSCs shared 183 significantly downregulated genes in *SIRT6*-deficient hMSCs compared with WT hMSCs. NRF2 target genes shared in EP and LP were indicated. **(D)** RT-qPCR analysis of NRF2 target genes in WT and *SIRT6*-deficient hMSCs. Values were normalized against 18S rRNA. Data were presented as mean ± SEM, *n* = 3, *P < 0.05, **P < 0.01, ***P < 0.001. **(E)** Average profile of the H3K4me3 histone modification around the gene body regions of NRF2 target genes in *SIRT6*-deficient and WT hMSCs. TSS, transcription start site; TTS, transcription termination site. **(F**, **G)** RT-qPCR **(F)** and western blotting **(G)** analyses of HO-1 expression in WT and *SIRT6*-deficient hMSCs treated with 25 μM PX-12 for the indicated times. Relative mRNA and protein expressions were presented as fold induction. For RT-qPCR **(F)**, values were normalized against 18S rRNA. Data were presented as mean ± SEM, *n* = 3, *P < 0.05, **P < 0.01. **(H)** Overexpression of SIRT6 (WT), not SIRT6 (HY), in *SIRT6*-deficient hMSCs partially restored HO-1 transcript. Values were normalized against 18S rRNA. Data were presented as mean ± SEM, *n* = 3, **P < 0.01, ***P < 0.001.

To uncover the molecular mechanism underlying how SIRT6 regulates redox homeostasis in hMSCs, we performed genome-wide RNA sequencing (RNA-seq) and H3K4me3 chromatin immunoprecipitation (ChIP) sequencing (ChIP-seq) analyses. We identified 119 upregulated genes and 246 downregulated genes in *SIRT6*^−/−^ hMSCs relative to WT hMSCs (Figure 3A and Supplementary information, Figure S3A–S3C and Tables S1–S2). We analyzed biological pathways that were potentially altered due to *SIRT6* deficiency and found that *SIRT6*^−/−^ hMSCs had specific downregulation in genes associated with the GO terms such as “skeletal system development” (Figure 3B and Supplementary information, Figure S3D), which is in agreement with observed phenotypic defects in osteogenesis and chondrogenesis (Supplementary information, Figure S1G). Interestingly, the most significant GO term for downregulated genes was “response to oxygen levels” (*P* = 4.8E−8), which included a list of NRF2-regulated antioxidant genes^16,40^ (Figure 3C and 3D and Supplementary information, Figure S3D and S3E and Table S3). These genes were markedly downregulated in *SIRT6*^−/−^ hMSCs (Figure 3D), which was associated with decreased levels of active chromatin mark H3K4me3 at their promoters (Figure 3E and Supplementary information, Figure S3F and S3G). In contrast to previous studies in mouse cells^23,24,25,41^, we did not observe global upregulation of HIF1α, c-MYC, c-JUN, and NF-κB target genes in *SIRT6*^−/−^ hMSCs (Supplementary information, Figures S4A and S4B). In addition, SIRT6 deficiency-induced coordinated downregulation of NRF2 target genes appears to be specific to hMSCs, as we did not observe this in *SIRT6*^−/−^ human vascular endothelial cells (hVECs) differentiated from *SIRT6*^−/−^ hESCs (Supplementary information, Figure S5A–S5C).

Among the NRF2-responsive genes downregulated in *SIRT6*^−/−^ hMSCs, we chose to focus on HO-1 (also referred to as HMOX1), which encodes a potent antioxidative and cytoprotective factor^42,43,44^. We found that HO-1 was quickly induced by PX-12 treatment with mRNA level peaking at 4 h and protein level peaking at 8 h in WT hMSCs (Figure 3F and 3G), indicating that HO-1 is an early response gene induced by oxidative stress in hMSCs. In contrast, HO-1 expression level in *SIRT6*^−/−^ hMSCs was insensitive to PX-12 treatment (Figure 3F and 3G), which was partially restored by overexpression of WT SIRT6, but not the H133Y mutant (Figure 3H). These data indicate that SIRT6 is required for the induction of HO-1 in response to oxidative stress.

**Figure 4:**
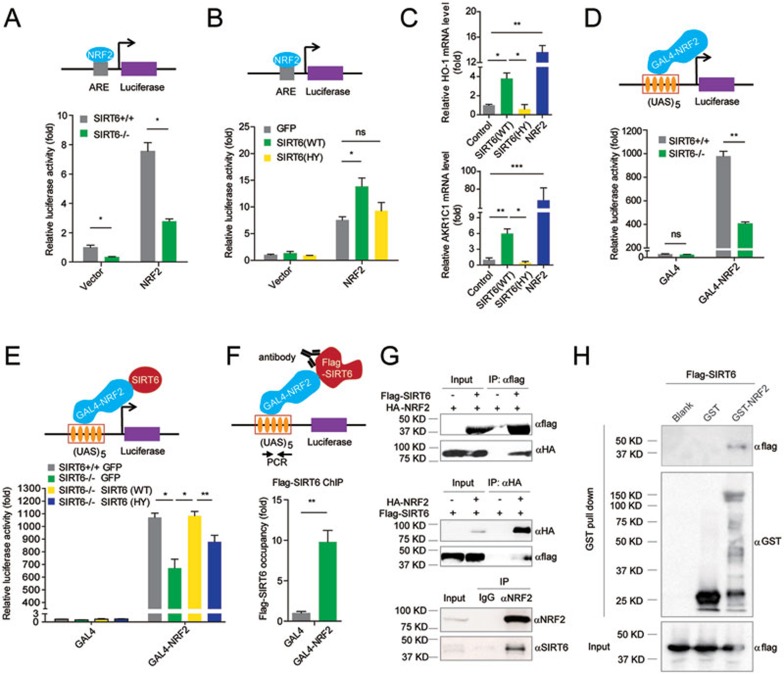
SIRT6 interacts with NRF2 and positively regulates NRF2-ARE pathway. **(A)** Transcriptional activity of NRF2 in WT and *SIRT6*-deficient hMSCs was measured by ARE-driven luciferase reporter assay. WT and *SIRT6*-deficient hMSCs were transfected with pcDNA3.1 (vector) or pcDNA3.1-NRF2 (NRF2), together with ARE-luciferase and Renilla plasmids. Data were presented as mean ± SEM, *n* = 3, *P < 0.05. **(B)** Plasmid expressing GFP, SIRT6 (WT), or SIRT6 (HY) was transfected into hMSCs, together with NRF2 or vector, and then NRF2 activity was measured using ARE-driven luciferase reporter. Data were presented as mean ± SEM, *n* = 3, NS, not significant, *P < 0.05. **(C)** Effect of SIRT6 overexpression on activation of NRF2 target genes in primary hMSCs. hMSCs were transduced with luciferase (control), SIRT6 (WT), SIRT6 (HY), or NRF2, and then the HO-1 and AKR1C1 transcripts were determined by RT-qPCR. Data were presented as mean ± SEM, *n* = 3, *P < 0.05, **P < 0.01, ***P < 0.001. **(D)** WT and *SIRT6*-deficient hMSCs were transfected with a (UAS)_5_-TATA-luciferase plasmid together with GAL4 or GAL4-NRF2, and then the NRF2 transactivity was measured. Data were presented as mean ± SEM, *n* = 3, NS, not significant, **P < 0.01. **(E)** Luciferase analysis of NRF2 transactivity in WT and *SIRT6*^−/−^ hMSCs in the presence of overexpressed GFP, SIRT6 (WT), or SIRT6 (HY). Data were presented as mean ± SEM, *n* =3, *P < 0.05, **P < 0.01. **(F)** ChIP-qPCR analysis of (UAS)_5_-associated SIRT6 in hMSCs co-expressing (UAS)_5_-TATA-luciferase, GAL4-NRF2, and Flag-SIRT6 using an anti-Flag antibody. Data were presented as mean ± SEM, *n* = 3, **P < 0.01. **(G)** Co-immunoprecipitation (Co-IP) showing that SIRT6 and NRF2 formed a protein complex. Exogenous (upper and middle panels) and endogenous (lower panel) Co-IPs were performed with the indicated antibodies. **(H)** GST-NRF2 or GST protein expressed from *E. coli* was incubated with Flag-SIRT6 expressed from HEK293T cells. The GST pull-down assay indicated an *in vitro* interaction between NRF2 and SIRT6.

**Figure 5:**
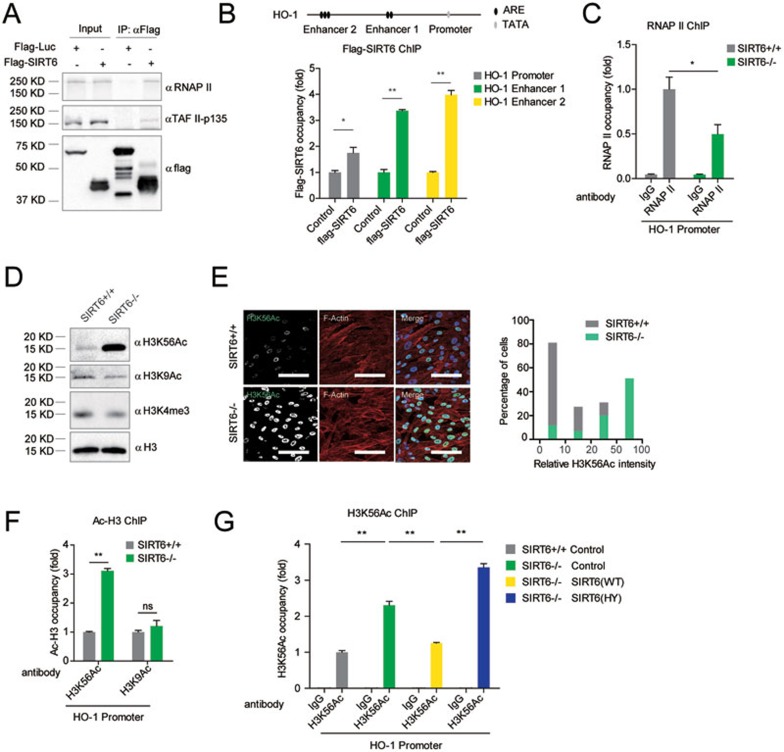
SIRT6 deacetylates H3K56 and is required for recruiting RNAP II to the HO-1 gene promoter. **(A)** Co-IP assay using protein extracts from HEK293T cells expressing Flag-SIRT6 indicated that SIRT6 formed a protein complex with RNAP II and TAF II-p135. **(B)** ChIP-qPCR performed in *SIRT6*^−/−^ hMSCs transduced with Flag-SIRT6 or Flag-luciferase (control) indicated association of SIRT6 with HO-1 promoter and enhancers. Data were presented as mean ± SEM, *n* = 3, *P < 0.05, **P < 0.01. **(C)** ChIP-qPCR assay showing SIRT6-dependent recruitment of RNAP II at HO-1 promoter. Data were presented as mean ± SEM, *n* = 3, *P <0.05. **(D)** Western blotting analyses of H3K56Ac, H3K9Ac, and H3K4me3 in WT and *SIRT6*-deficient hMSCs. Histone 3 (H3) was used as the loading control. **(E)** Immunofluorescence (left) and statistical (right) analyses of H3K56Ac levels in WT and *SIRT6*-deficient hMSCs. Scale bar, 100 μm. **(F)** ChIP-qPCR analysis of the enrichment of H3K56Ac and H3K9Ac at HO-1 promoter in WT and *SIRT6*-deficient hMSCs. Data were presented as mean ± SEM, *n* = 3, NS, not significant, **P < 0.01. **(G)** ChIP-qPCR analysis of H3K56Ac at HO-1 promoter in WT or *SIRT6*-deficient hMSCs transduced with lentiviral vector encoding SIRT6 (WT), SIRT6 (HY), or luciferase (control). Data were presented as mean ± SEM, *n* = 3, **P < 0.01.

We next determined whether SIRT6 is directly involved in the NRF2-mediated transcription. We transfected *SIRT6*^−/−^ and WT hMSCs with an ARE-driven luciferase reporter. Compared with WT, SIRT6 deficiency resulted in a significantly reduced luciferase activity in hMSCs (Figure 4A). Overexpression of WT SIRT6, but not its H133Y mutant, enhanced NRF2-mediated gene expression in primary hMSCs (Figure 4B and 4C), suggesting that SIRT6 positively regulated NRF2-mediated transcription in a deacetylase-dependent manner. There was no discernible difference in nuclear NRF2 levels between WT and *SIRT6*^−/−^ hMSCs (Supplementary information, Figure S5D). ChIP-qPCR analysis also indicated that SIRT6 deficiency did not affect the amount of NRF2 associated with the classic ARE of endogenous HO-1 enhancer (Supplementary information, Figure S5E). These observations raised an intriguing possibility that SIRT6 directly coactivated NRF2-mediated transcription. To test this, we used the GAL4(DBD)-NRF2/(UAS)_5_-TATA-luciferase system. We found that the transactivation ability of NRF2 was positively regulated by SIRT6 in a deacetylase-dependent manner (Figure 4D and 4E). Using this system, we also identified that NRF2 was able to mediate recruitment of SIRT6 to the promoter DNA, the (UAS)_5_, GAL4-binding site by ChIP-qPCR (Figure 4F). Co-immunoprecipitation (Co-IP) and GST pull-down analyses showed that SIRT6 associated with NRF2 in a protein complex (Figure 4G, 4H and Supplementary information, Figure S5F and S5G).We next examined whether SIRT6 serves as a bridge between NRF2 and the basal transcriptional machinery. Co-IP experiment showed that SIRT6 formed a protein complex with RNA polymerase II (RNAP II) and TAF II-p135, two components of RNAP II complex (Figure 5A). ChIP-qPCR analysis further identified that both SIRT6 and RNAP II were recruited to the HO-1 promoter in WT hMSCs (Figure 5B and 5C). SIRT6 deficiency led to reduction of HO-1 promoter-bound RNAP II (Figure 5C). These data suggest that SIRT6 could help assemble NRF2-RNAP II transcription complex at the HO-1 promoter.

### Deacetylation of H3K56 by SIRT6 accounts for recruitment of RNAP II to HO-1 promoter

In murine studies, SIRT6 was shown to act as a transcriptional repressor by deacetylating H3K9 at the gene promoters^23,24,25^. As SIRT6 deficiency did not result in upregulation of the H3K9Ac level, it is thus unlikely that H3K9Ac is the major substrate of SIRT6 in hMSCs (Figure 5D and Supplementary information, Figure S6A). In contrast, the level of H3K56Ac, another substrate of SIRT6^28,45^, was dramatically increased in *SIRT6*^−/−^ hMSCs (Figure 5D, 5E and Supplementary information, Figure S6A). In addition, ChIP-qPCR showed more enrichment of acetylated H3K56 at the promoters of NRF2-responsive genes including HO-1, AKR1C1, and PTGS2 in *SIRT6*^−/−^ than in WT hMSCs (Figure 5F and Supplementary information, Figure S6B). We observed a reduced H3K56 acetylation level at HO-1 promoter when WT SIRT6 but not the H133Y mutant was reintroduced into *SIRT6*^−/−^ hMSCs (Figure 5G and Supplementary information, Figure S6C). In addition, reintroduction of WT SIRT6 instead of the H133Y mutant increased the recruitment of RNAP II to the HO-1 promoter in *SIRT6*^−/−^ hMSCs (Supplementary information, Figure S6D), suggesting a deacetylase activity-dependent recruitment of RNAP II by SIRT6. Next, we investigated whether deacetylation of H3K56 by SIRT6 at HO-1 promoter is causally linked to the recruitment of RNAP II. Ectopic expression of H3K56Q, an H3K56 acetylation-mimic mutant^28^, in WT hMSCs inhibited the recruitment of RNAP II to the HO-1 promoter (Supplementary information, Figure S6E). On the other hand, ectopic expression of H3K56R, an acetylation-defective mutant^46^, in *SIRT6*^−/−^ hMSCs promoted occupancy of RNAP II at HO-1 promoter (Supplementary information, Figure S6E). Altogether, these data indicated that SIRT6 is responsible for the deacetylation of H3K56Ac at the HO-1 promoter in hMSCs, which is important for the recruitment of RNAP II transcriptional machinery.

### SIRT6-NRF2-HO-1 pathway accounts for anti-oxidative response in hMSCs

**Figure 6:**
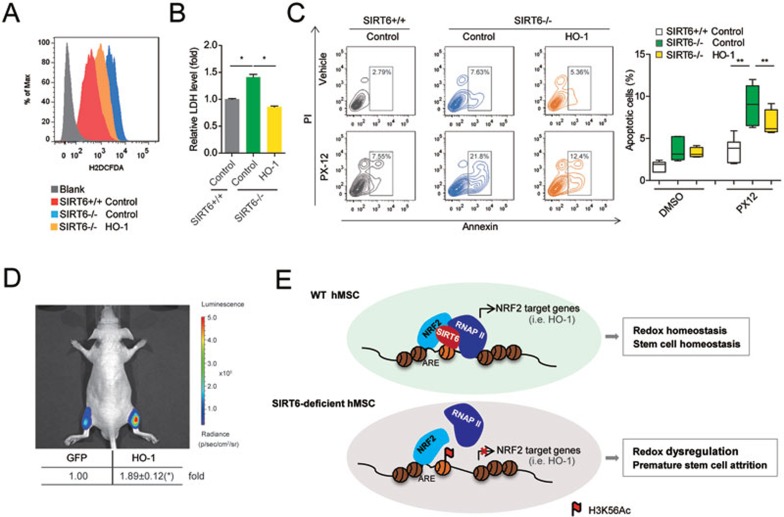
Compromised NRF2-HO-1 axis accounts for redox dysregulation in *SIRT6*-deficient hMSCs. **(A)** FACS analyses of ROS level in hMSCs transduced with lentiviral vector encoding luciferase (control) or HO-1. **(B)** Lactate dehydrogenase (LDH) detection in the indicated hMSCs transduced with lentiviral vector encoding luciferase (control) or HO-1 in the presence of 50 μM PX-12 treatment. Data were presented as mean ± SEM, *n* = 3, *P < 0.05. **(C)** FACS analyses of PX-12-induced cytotoxicity in hMSCs transduced with lentiviral vector encoding luciferase (control) or HO-1 (left panel). Cells were treated with vehicle (DMSO) or 50 μM PX-12 for 24 h. Statistical analysis of apoptotic cells (right panel) was presented as mean ± SEM. *n* = 6, **P < 0.01. **(D)** Measurement of luciferase activity in immunodeficient mice with IVIS. *SIRT6*-deficient hMSCs overexpressing GFP plus luciferase (control group, left) and *SIRT6*-deficient hMSCs overexpressing HO-1 plus luciferase (right) were implanted into the TA muscles of mice. Six days after implantation, mice were intraperitoneally injected with 20 mg/kg PX-12 for 24 h, and then luciferase activity was measured. Data were presented as mean ± SEM, *n* = 4, *P < 0.05. **(E)** A putative model for SIRT6-mediated redox regulation in hMSCs. In WT hMSCs, SIRT6 is a key regulator of the cellular redox homeostasis by co-activating NRF2 antioxidant pathway. SIRT6 associates with NRF2 and deacetylates H3K56 at the promoter of NRF2 target genes (i.e., HO-1), which is required for the recruitment of RNAP II complex and subsequent transactivation of NRF2. In *SIRT6*-deficient hMSCs, SIRT6 deficiency causes increased level of H3K56Ac and impaired recruitment of RNAP II complex to HO-1 promoter, resulting in decrease in HO-1 expression and compromised cellular redox homeostasis.

As *SIRT6*^−/−^ hMSCs exhibited increased basal ROS production and increased sensitivity to oxidative stress, next we tested whether the SIRT6-NRF2-HO-1 axis is linked to hMSCs' anti-oxidative response. We found that overexpression of HO-1 resulted in alleviation of ROS levels in *SIRT6*^−/−^ hMSCs (Figure 6A). In addition, we observed a decrease in the susceptibility of *SIRT6*^−/−^ hMSCs to PX-12-induced cytotoxicity upon HO-1 overexpression (Figure 6B and 6C). Finally, we investigated whether SIRT6-NRF2-HO-1 axis functions in the regulation of hMSC redox homeostasis in an *in vivo* context. To this end, *SIRT6*^−/−^ hMSCs were transduced with a lentiviral HO-1 expression vector and then transplanted into the TA muscles of immunodeficient mice (Figure 6D and Supplementary information, Figure S6F). As expected, HO-1 overexpression repressed accelerated cell attrition of *SIRT6*-deficient hMSCs in an *in vivo* microenvironment (Figure 6D).

## Disscusion

Throughout lifetime, tissue stem cells are constantly exposed to various stresses including ROS, toxins, and chemical or physical stressors, which may result in premature cellular aging or decreased cellular survival. Here, by employing the state-of-the-art genome-editing technology to specifically delete *SIRT6* in human stem cells, we have presented several lines of evidence in support of a role of SIRT6 in safeguarding hMSCs from functional decay: (1) SIRT6 deficiency leads to an increase in ROS levels and vulnerability to oxidative insults; (2) *SIRT6*-depleted hMSCs show accelerated cellular senescence; (3) SIRT6 deficiency results in impairment of differentiation potential of hMSCs into bone and cartilage. These new findings establish a previously unappreciated connection between SIRT6, oxidative stress, and human stem cell exhaustion, and indicate that SIRT6-NRF2 module may be the key molecular hub to protect the mesodermal tissues from aging-associated degeneration.

Mechanistically, SIRT6 is identified as a positive regulator of NRF2-ARE antioxidant pathway in hMSCs (Figure 6E). We propose a model where SIRT6 plays dual roles in NRF2-mediated HO-1 transcription: (1) SIRT6 stabilizes the protein complex comprised of NRF2 and basal transcription machinery, potentially as an adaptor protein; (2) SIRT6 also exerts its histone deacetylase activity specifically at the promoter of HO-1. The two functions of SIRT6 are potentially linked as SIRT6-mediated deacetylation of H3K56 may be a prerequisite for stabilizing the NRF2-SIRT6-RNAP II complex at chromosomal levels.

To the best of our knowledge, this study provides the first evidence that SIRT6 can serve as a transcriptional coactivator. Recent murine studies showed that SIRT6 could transrepress the activities of HIF1α, c-JUN, and NF-κB via H3K9 deacetylation^23,24,25^. In hMSCs, however, we found that acetylated H3K9 is not a preferred substrate for SIRT6. Likewise, we did not observe global upregulation of the target genes of HIF1α, c-MYC, c-JUN, and NF-κB^23,24,25,41^ in *SIRT6*-depleted hMSCs. Instead, deficiency of SIRT6 in hMSCs results in an increase in acetylated H3K56 levels, and SIRT6-mediated deacetylation of H3K56 is a key event facilitating NRF2-dependent gene expression. In addition, in line with a role of SIRT6 as a NRF2 coactivator, SIRT6 interacts with RNAP II complex and the depletion of SIRT6 in hMSCs diminishes the enrichment of RNAP II complex at the HO-1 promoter. Of note, a recent genome-wide study in hESCs revealed that SIRT6 co-localizes with RNAP II at gene promoter regions^47^, which together with our finding raises an interesting possibility that other SIRT6-mediated transcriptional activation events may also exist. In addition, it should be noted that SIRT6-mediated positive regulation of NRF2 activity appears to be specific to hMSCs. Indeed, we did not observe global downregulation of NRF2 target genes in *SIRT6*-deficient hVECs. In these contexts, more complex regulations such as cell-type-specific protein-protein interactions, deacetylation-independent activities of SIRT6, as well as potential compensations from other histone deacetylases may be involved.

The implication of SIRT6-regulated H3K56 acetylation in various cellular events has not been fully investigated. A very recent study indicated that human SIRT6 acts as a scaffold for recruiting SNF2H, a chromatin remodeler, and other DNA repair factors to DNA damage foci, an event dependent on SIRT6's deacetylase activity at histone H3K56^28^. It is likely that SIRT6-mediated low acetylation level of H3K56 at DNA damage site is important for an open local chromatin configuration necessary for DNA repair. Along this line of thinking, one could reason that transcriptional events are also aided by a permissible chromatin configuration, i.e., chromatin remodeling-dependent factor recruitment. Therefore, it is reasonable that SIRT6/NRF2-dependent HO-1 transcription may employ a similar H3K56 deacetylation-based mechanism to recruit RNAP II complex. How the increase in local H3K56Ac levels potentially regulates chromosomal configuration and recruitment of transcriptional machineries warrants further investigation.

The findings that SIRT6-NRF2-HO-1 axis acts as a regulator for stem cell redox homeostasis and SIRT6 functions as a transcriptional coactivator undoubtedly add a new layer of knowledge to SIRT6 biology. Safeguarding redox homeostasis by SIRT6 in hMSCs principally could contribute to mechanistic explanations for various SIRT6-related biological processes, including genomic instability, cellular senescence, cellular transformation, and metabolic dysregulation. Our study provides first evidence that SIRT6 is a key gatekeeper for human adult stem cell homeostasis, highlighting that SIRT6-NRF2 pathway may be a novel target for preventing aging-associated stem cell attrition, and hopefully, for treating aging-associated disorders in the future.

## Materials and Methods

### Cell culture

H9 hESCs (WiCell Research) were maintained on Mitomycin C-inactivated mouse embryonic fibroblast (MEF) or Matrigel^31,48^. hMSCs were cultured in DMEM (Hyclone) medium supplemented with 10% fetal bovine serum (FBS, AusGeneX), 0.1 mM non-essential amino acids (Gibco), 1% penicillin/streptomycin (Gibco), and 1 ng/ml bFGF (Joint Protein Central, JPC)^31^. Human primary MSCs were isolated from the teeth tissue of a 13-year-old male person and were cultured in hMSC culture medium. hVECs were cultured in EGM-2 (Lonza) medium containing VEGF165 (HumanZyme, 50 ng/ml) and FGF2 (JPC, 20 ng/ml).

### Generation of *SIRT6*^−/−^ hESCs

TALEN-mediated gene targeting was performed as previously described^31^. In brief, a donor plasmid was constructed by the combination of 1.0-2.0-kb homology arms and drug resistance cassettes (neo). H9 hESCs (5.0 × 10^6^) were electroporated with a pair of TALEN vectors (Addgene, TAL2454 (Plasmid #36843) and TAL2455 (Plasmid #36844)) and the donor plasmid, and subsequently cultured on MEF feeder. G418 (100 μg/ml, Invitrogen) was then added to the medium to initiate positive selection 2-4 days after electroporation. After about 2 weeks' selection, G418-resistant clones were picked and transferred to a 96-well plate for further characterization and expansion. Gene-targeted clones were determined by genomic PCR with the primers listed in Supplementary information, Table S4 using PrimeSTAR DNA Polymerase (TAKARA). Long PCR cycling included a 1-min initial denaturation at 98 °C, 35 cycles of 10-s denaturation at 98 °C and a 0.5-3-min annealing and extension at 68 °C plus a final extension at 72 °C for 7 min.

### Southern blotting

Genomic DNA was extracted following a described strategy^48^. 20 μg genomic DNA of each samples was digested with *Hin*d III and *Xho* I (NEB) overnight and subjected to electrophoresis at 50 V on a 0.8% agarose gel (SeaKem Gold agarose, Lonza) for 3-4 h. The gel was subsequently incubated in 0.25 M HCl for 3-5 min followed by 2× 15-min incubation in denaturation buffer (0.5 M NaOH, 1.5 M NaCl) and 2× 15-min incubation in neutralization buffer (0.5 M Tris-HCl, pH 7.5; 1.5 M NaCl). The DNA was then blotted overnight onto a nylon membrane (GE Healthcare Life Sciences) by capillary transfer in 20× SSC buffer. The membrane was then ultraviolet crosslinked. The 5′ and 3′ probes were amplified from genomic DNA using a DIG-label kit (Roche) with the primers in Supplementary information, Table S4, following the manufacturer's protocol. The probes were labeled with DIG and Southern hybridization was performed following the standard protocol.

### Excision of the neomycin-resistance cassette

To remove the neomycin-resistance cassette, *SIRT6* gene-targeted hESCs were electroporated with pCAG-FLpo-2A-puro vector and then cultured on MEF feeder. Three days after transfection, puromycin (1 μg/ml; Invitrogen) was used to enrich puro-resistant cells. Puromycin was withdrawn after 48 h. Ten days later, the emerging colonies were picked and expanded in 96-well plates. Removal of the neomycin-resistance cassette was verified by PCR using PrimeSTAR DNA Polymerase (TAKARA) with the related primers (Supplementary information, Table S4).

### hMSC generation and characterization

hMSCs were differentiated from hESCs based on a published protocol^31^. Briefly, embryoid bodies were left to differentiate in αMEM (Invitrogen) medium supplemented with 10% FBS (AusGeneX), 10 ng/ml bFGF (JPC), 5 ng/ml TGFβ (HumanZyme) and 1% penicillin/streptomycin (Gibco) until fibroblast-like cells appeared. The hMSCs were purified with different antibodies corresponding to hMSC-specific markers (CD73, CD90, and CD105) by FACS. Antibodies used for hMSC characterization were as follows: anti-CD105-APC (17-1057-42) antibody was purchased from eBioscience; anti-CD90-FITC (555595), anti-CD73-PE (550257), anti-CD34-PE (555822), anti-CD43-APC (580198), and anti-CD45-FITC (555482) antibodies were purchased from BD Biosciences. Anti-IgG-FITC (555748), anti-IgG-PE (555749), and anti-IgG-APC (555751) antibodies from BD Biosciences were used as isotype controls. The functionality of hMSC was further verified by differentiation towards cartilage, bone, and adipocytes^31^. The tri-lineage differentiation abilities of hMSC lines were evaluated by histochemical staining with von Kossa (osteogenesis), Alcian blue (chondrogenesis), and Oil red O (adiopogenesis) Kit (IHC World), respectively.

### Directed differentiation of hESCs into hVECs

Differentiation was performed as previously described^49^, and the generated hVECs were characterized by the expression of hVEC-specific markers VE-cadherin, CD31, and VWF, as well as the activity of AC-LDL uptake.

### Lentivirus preparation

The cDNAs of flag-SIRT6, flag-HO-1, flag-NRF2, Flag-luciferase, and flag-H3 were cloned into pLE4 lentiviral vector (a gift from Dr Tomoaki Hishida). pLE4-flag-SIRT6 (H133Y), pLE4-flag-H3K56Q, and pLE4-flag-H3K56R were generated using a fast mutagenesis kit (TransGen Biotech). Lentivirus particles were generated from HEK293T cells^48^, and used for transducing hMSCs in the presence of 4 μg/ml polybrene.

### SA-β-GAL staining assay

Cells were stained using SA-β-GAL assay according to a previously described method^48,50^.

### Clonal expansion assay

2 000 cells were seeded in each well of 12-well plates, cultured for 2 weeks, and stained with 0.2% crystal violet. Cell numbers were counted using light microscope in randomly selected fields. Each experiment was performed in triplicate.

### Cell viability analysis

hMSCs (2 × 10^4^) were seeded onto 0.1% gelatin-coated 96-well plates (Corning, Costar), and upon reaching 90% confluence cells were treated with various stressors for 24 h. Cell viability was measured using MTS approach according to the recommended protocol for CellTiter 96 AQueous One Solution Cell Proliferation Assay (Promega). Stressors used in this assay include DMSO (vehicle, Sigma), PX-12 (Santa Cruz Biotech), 4-nitroquinoline *N*-oxide (4NQO, Sigma), apoptosis activator 2 (AAT2, TOCRIS), ABT-737 (Santa Cruz Biotech), Mitomycin C (MMC, Sigma), and Stat3 inhibitor III (WP-1066, Santa Cruz), Paraquat (Sigma), NAC (Sigma).

### Cell death analysis

Cell death was determined using Cytotox 96 non-radioactive cytotoxicity assay (Promega). Cells were treated with DMSO or 50 μM PX-12 for 24 h, and then the LDH level in culture medium was assayed. Each experiment was performed in biological triplicate.

### Western blotting

Protein quantification was performed using a BCA Kit. Protein lysate was subjected to SDS-PAGE and subsequently electrotransferred to a polyvinylidene fluoride membrane (Millipore). Western blotting was performed as previously described^8,48,51^. The antibodies used are listed as follows (company, catalogue number, and dilution): anti-NRF2 (Abcam, ab62352, 1:1 000), anti-SIRT6 (Cell Signaling Technology, 12486, 1:1 000), anti-H3K9Ac (Millipore, 06-942, 1:1 000), anti-H3K56Ac (Abcam, ab76307, 1:5 000), anti-H3K4me3 (Active motif, 39159, 1:1 000), anti-P16 (Cell Signaling Technology, 4824, 1:200), anti-P21 (Santa Cruz, ZS-6246, 1:1 000), anti-γH2AX (Abcam, ab11175, 1:1 000), anti-HO-1 (Enzo, ADI-SPA-895-D, 1:1 000), anti-RNAP II (Covance, MMS-126R, 1:1 000), anti-β-actin (Santa Cruz, sc69879, 1:4 000), anti-lamin B1 (Abcam, ab16048, 1:1 000), anti-TAF II-p135 (Santa Cruz, sc136093, 1:1 000), anti-flag (Sigma, F1804, 1:4 000), and anti-HA (Santa Cruz, sc7392, 1:1 000).

### Immunofluorescence

hESCs and hMSCs were fixed with formaldehyde (4% in PBS) for 15 min, permeabilized with Triton X-100 (0.4% in PBS) for 15 min, incubated with blocking buffer (10% donkey serum in PBS) for 30 min, and stained with primary antibody overnight at 4 °C. Then, the cells were treated with secondary antibodies for 1 h at room temperature. Hoechst 33342 (Invitrogen) was used to stain nuclear DNA. The antibodies used in immunofluorescence assay are as follows: anti-SIRT6 (CST, 12486, 1:100), anti-NANOG (Abcam, ab21624, 1:200), anti-SOX2 (Santa Cruz, sc-17320, 1:100), anti-OCT4 (Santa Cruz, sc-5279, 1:100), anti-Flag (Sigma, F1804, 1:1 000), anti-SMA (Sigma, A5228, 1:100), anti-β-tubulin III (TUJ1, Sigma, T2200, 1:100), anti-FOXA2 (CST, 8186S, 1:100), anti-VE-caderin (Cell Signaling Technology, 2158, 1:100), anti-CD31 (BD Pharmingen, 555445, 1:50), anti-VWF (Dako, A0082, 1:200), anti-Ac-LDL (Life Technology, l23380, 1:400).

### Flow cytometry analysis

For cell cycle analysis, cells were pulsed with 10 μM EdU for 2 h, and collected using TrypLE Express (Invitrogen). Collected cells were stained with Alexa Fluor 647 dye azide and propidium iodide according to the manufacturer's instruction for Click-iT EdU Alexa Fluor 647 Flow Cytometry Assay Kit (Molecular Probes, c10419). Cell cycle profile was quantified by a FACS machine (BD LSRFortesa). For cell apoptosis analysis, cells were collected freshly and stained with Annexin V-EGFP and PI, and then apoptotic cells were quantified by FACS. For ROS measurement, cells were collected, loaded with 1 μM H2DCFDA for 30 min using ROS Detection Reagents (Molecular Probes, C6827), and quantified by FACS.

### Co-IP assay

For exogenous Co-IP, cells were lysed in NP-40 lysis buffer (1% NP-40, 150 mM NaCl, 10% glycerol, 1 mM EGTA, 1 mM EDTA, 20 mM Tris, pH 7.5, and complete protease inhibitor cocktail (Roche)), and for endogenous Co-IP, cells were lysed in CHAPS buffer (120 mM NaCl, 0.3% CHAPS, 1 mM EDTA, 40 mM HEPES, pH 7.5, and complete protease inhibitor cocktail (Roche)). Cells were lysed on ice for 30 min, and then centrifuged at 12 000 rpm. at 4 °C for 20 min. For endogenous Co-IP, lysates (1 mg protein) were pre-cleared with 20 μl of Protein A/G-PLUS Agarose beads (Santa Cruz) for 2-4 h, and then the supernatants were collected by centrifugation at 3 000 rpm at 4 °C for 3 min. The supernatants mixed with the indicated antibodies and beads were rotated overnight at 4 °C. After being washed with NP-40 buffer or CHAPS buffer for three times, the immunocomplexes were eluted by boiling in 1× SDS-loading buffer for 10 min.

### GST pull-down assay

Recombinant GST and GST-NRF2 proteins were expressed in BL21 cells (Genestar)^52^. Flag-SIRT6 protein was expressed in HEK293T. GST pull-down assay was performed as previously described^53^.

### Luciferase reporter assay

For ARE-driven luciferase reporter system, hMSCs were co-transfected with 0.15 μg of ARE-driven luciferase, 0.15 μg pcDNA3.1-NRF2, 0.15 μg of cDNA expression vector, and 0.05 μg of renilla. For GAL4(DBD)-NRF2/(UAS)_5_-TATA luciferase reporter system, cells were co-transfected with 0.15 μg of (UAS)_5_-TATA luciferase, 0.05 μg of renilla, 0.15 μg of cDNA expression vector, and 0.15 μg of GAL4-fusion protein construct. At 48 h after transfection, cells were collected and relative luciferase activity was measured using Dual-Luciferase Reporter Assay System (Promega).

### RNA-seq library construction

One million cells were applied to extract total RNA using the RNeasy Mini Kit (Qiagen) according to the manuals. After quantification of RNA by Fragment Analyzer (Advanced Analytical), 1.5 μg of total RNA was used to construct sequencing libraries by TruSeq RNA Sample Preparation Kit (Illumina) following the manufacturer's standard protocol.

### Genomic library construction

Genomic DNA was extracted from 0.5-1 × 10^6^ cells via DNeasy Blood & Tissue Kit (Qiagen). After being sheared into fragments ranging from 150 to 200 bp, the DNA was constructed into sequencing libraries using NEBNext DNA Library Prep Reagent Set for Illumina (NEB).

### ChIP-seq

ChIP-seq was performed according to a previous protocol with some modifications^54^. Briefly, cells were crosslinked and lysed, and then the released chromatin was sheared into fragments. To obtain fragments of interest, samples were incubated with anti-H3K4me3 antibody (Abcam, ab8580) overnight. Inputs served as negative controls. After the DNA was de-crosslinked and extracted, the ChIP-seq libraries were generated using NEBNext DNA Library Prep Reagent Set for Illumina (NEB) according to manuals.

### ChIP-seq data analysis

Clean reads were mapped to hg19 genome by BWA. Then, peaks were called by MACS2^55^ by default parameters using input as control. Density of the promoter region was defined as the non-duplicate reads located in the regions between 2-kb upstream of the TSS (transcription start site) and 2-kb downstream of the TSS and then were normalized by the total non-duplicate reads that were mapped to the hg19 genome.

### RNA-seq data analysis

Low-quality reads (N bases >10% of a read, >50% of the bases in a read with phred 33 < 5, or phred score of the first 5 bases < 20) were discarded. Then reads were mapped to the human reference genome hg19 (from UCSC) by Tophat^56^ and Cufflinks^57^. Transcript expression and differentially expressed genes were analyzed as previously reported^58^.

### CNV analysis

Clean reads of genomic libraries were first mapped to hg19 genome by BWA^59^. Then, the reads in 0.5-M window along the genome were normalized by the total bases mapped to the reference and the number of non-N bases in 0.5-M genome window size.

### RT-qPCR

Total RNA was extracted from cells according to the instruction of TRIzol Reagent (Invitrogen). 1-2 μg total RNA was used for cDNA synthesis with reverse transcription Master Mix (Promega). Quantitative real-time PCR was carried out using iTaq Universal SYBR Green Supermix (Bio-Rad) on a CFX384 Real-Time PCR system (Bio-Rad). All data were normalized by 18S rRNA transcript and calculated using the ΔΔCq method. All RT-qPCR primer pairs are listed in Supplementary information, Table S4.

### ChIP-qPCR

ChIP-qPCR was performed according to a published protocol^54^. The antibodies used in ChIP-qPCR were as follows: anti-NRF2 (Abcam, ab62352, 4 μl), anti-RNAP II (Covance, MMS-126R, 4 μl), anti-H3K9Ac (Millipore, 06-942, 4 μl), anti-H3K56Ac (Abcam, ab76307, 2 μl), anti-flag (Sigma, F1804, 2.4 μl). The primers' sequences are listed in Supplementary information, Table S4.

### Animal experiments

For mouse experiments, 6-8-week-old male NOD-SCID mice and immune-deficient CD-1 nude mice were used. Animal experiments performed in this study were approved by the Chinese Academy of Science Institutional Animal Care and Use Committee. For teratoma formation assay, NOD-SCID mice were injected s.c. with 3 × 10^6^ WT and *SIRT6*-deficient hESCs in a Matrigel/mTeSR solution, respectively. Teratomas were collected when reaching a size of around 10 mm in diameter, and subjected to immunostaining. For hMSC transplantation assay, 10^6^ WT and *SIRT6*-deficient hMSCs pretransduced with a lentiviral vector encoding luciferase were injected to TA muscle of CD-1 nude mice. Mice were imaged *in vivo* with IVIS spectrum imaging system (XENOGEN, Caliper) for luciferase activity. After imaging, TA muscle of mice was subjected to immunofluorescence analysis.

### Statistical analysis

Results were presented as mean ± SEM. Two tailed Student's *t*-test was conducted using Graph-Pad Prism Software. *P* values < 0.05 were considered statistically significant (*).

### Accession numbers

All of the RNA-seq and ChIP-seq data have been deposited in GEO under the accession number GSE64642. Whole-genome sequencing data for CNV analysis were deposited in SRA under the accession number SRP059859.

## Author Contributions

HP, DG, and XL performed the majority of the experiments. HP performed the gene editing. DG and HP performed cell culture, differentiation, and cell functional analysis. LW, JQ, TY, JY, WZ, and GY performed cell culture and differentiation. FT, XL, and JL carried out the genomic and epigenomic analyses. HP and JZ performed the co-IP experiments. HP, DG, RR, WZ, YL, JY, YH, and HW performed animal experiments. JW, WZ, ZJ, ZM, and JL performed data analysis and wrote the manuscript. GHL, FT, JQ, HP, DG, and XL conceived this study and wrote the manuscript.

## Competing Financial Interests

The authors declare no competing financial interests.

## Supplementary Information

Supplementary information, Figure S1 Characterization of SIRT6-deficient hESCs and differentiation of hESCs to hMSCs.

Supplementary information, Figure S2 Characterization of SIRT6-deficient hMSCs.

Supplementary information, Figure S3 Gene expression analyses of WT and SIRT6-deficient hMSCs.

Supplementary information, Figure S4 Gene expression and epigenome analyses of WT and SIRT6-deficient hMSCs.

Supplementary information, Figure S5 SIRT6 regulates recruitment of RNAP II to HO-1 promoter in a deacetylase-dependent manner.

Supplementary information, Figure S6 HO-1 overexpression rescues premature attrition of SIRT6-deficient hMSCs *in vivo*.

Supplementary information, Table S1 Up- and downregulated genes in *SIRT6*^−/−^ hMSCs relative to WT hMSCs (early passage).

Supplementary information, Table S2 Up- and downregulated genes in *SIRT6*^−/−^ hMSCs relative to WT hMSCs (late passage).

Supplementary information, Table S3 GO terms for downregulated genes in *SIRT6*^−/−^ hMSCs.

Supplementary information, Table S4 Sequences of primers and probes used in the study.

## References

[bib1] 1Lopez-Otin C, Blasco MA, Partridge L, Serrano M, Kroemer G. The hallmarks of aging. Cell 2013; 153:1194–1217. 23746838 10.1016/j.cell.2013.05.039 PMC3836174

[bib2] 2Liu L, Cheung TH, Charville GW, et al. Chromatin modifications as determinants of muscle stem cell quiescence and chronological aging. Cell Rep 2013; 4:189–204. 23810552 10.1016/j.celrep.2013.05.043 PMC4103025

[bib3] 3Uccelli A, Moretta L, Pistoia V. Mesenchymal stem cells in health and disease. Nat Rev Immunol 2008; 8:726–736. 19172693 10.1038/nri2395

[bib4] 4Lepperdinger G. Inflammation and mesenchymal stem cell aging. Curr Opin Immunol 2011; 23:518–524. 21703839 10.1016/j.coi.2011.05.007 PMC3167021

[bib5] 5Liang R, Ghaffari S. Stem cells, redox signaling, and stem cell aging. Antioxid Redox Signal 2014; 20:1902–1916. 24383555 10.1089/ars.2013.5300 PMC3967383

[bib6] 6Stenderup K, Justesen J, Clausen C, Kassem M. Aging is associated with decreased maximal life span and accelerated senescence of bone marrow stromal cells. Bone 2003; 33:919–926. 14678851 10.1016/j.bone.2003.07.005

[bib7] 7Liu GH, Barkho BZ, Ruiz S, et al. Recapitulation of premature ageing with iPSCs from Hutchinson-Gilford progeria syndrome. Nature 2011; 472:221–225. 21346760 10.1038/nature09879 PMC3088088

[bib8] 8Liu GH, Qu J, Suzuki K, et al. Progressive degeneration of human neural stem cells caused by pathogenic LRRK2. Nature 2012; 491:603–607. 23075850 10.1038/nature11557 PMC3504651

[bib9] 9Yu KR, Kang KS. Aging-related genes in mesenchymal stem cells: a mini-review. Gerontology 2013; 59:557–563. 23970150 10.1159/000353857

[bib10] 10Zhang WQ, Li JY, Suzuki K, et al. A Werner syndrome stem cell model unveils heterochromatin alterations as a driver of human aging. Science 2015; 348:1160–1168. 25931448 10.1126/science.aaa1356 PMC4494668

[bib11] 11Lavasani M, Robinson AR, Lu AP, et al. Muscle-derived stem/progenitor cell dysfunction limits healthspan and lifespan in a murine progeria model. Nat Commun 2012; 3:608. 22215083 10.1038/ncomms1611 PMC3272577

[bib12] 12Singh L, Brennan TA, Kim JH, et al. Brief report: long-term functional engraftment of mesenchymal progenitor cells in a mouse model of accelerated aging. Stem Cells 2013; 31:607–611. 23193076 10.1002/stem.1294 PMC3582822

[bib13] 13Paul MK, Bisht B, Darmawan DO, et al. Dynamic changes in intracellular ROS levels regulate airway basal stem cell homeostasis through Nrf2-dependent Notch signaling. Cell stem cell 2014; 15:199–214. 24953182 10.1016/j.stem.2014.05.009 PMC4127166

[bib14] 14Moore KA, Lemischka IR. Stem cells and their niches. Science 2006; 311:1880–1885. 16574858 10.1126/science.1110542

[bib15] 15Ray PD, Huang BW, Tsuji Y. Reactive oxygen species (ROS) homeostasis and redox regulation in cellular signaling. Cell Signal 2012; 24:981–990. 22286106 10.1016/j.cellsig.2012.01.008 PMC3454471

[bib16] 16Gorrini C, Harris IS, Mak TW. Modulation of oxidative stress as an anticancer strategy. Nat Rev Drug Discov 2013; 12:931–947. 24287781 10.1038/nrd4002

[bib17] 17Bailey-Downs LC, Mitschelen M, Sosnowska D, et al. Liver-specific knockdown of IGF-1 decreases vascular oxidative stress resistance by impairing the Nrf2-dependent antioxidant response: a novel model of vascular aging. J Gerontol A Biol Sci Med Sci 2012; 67:313–329. 22021391 10.1093/gerona/glr164 PMC3309870

[bib18] 18Valcarcel-Ares MN, Gautam T, Warrington JP, et al. Disruption of Nrf2 signaling impairs angiogenic capacity of endothelial cells: implications for microvascular aging. J Gerontol A Biol Sci Med Sci 2012; 67:821–829. 22219515 10.1093/gerona/glr229 PMC3403863

[bib19] 19Mahlknecht U, Ho AD, Voelter-Mahlknecht S. Chromosomal organization and fluorescence *in situ* hybridization of the human Sirtuin 6 gene. Int J Oncol 2006; 28:447–456. 16391800

[bib20] 20Mostoslavsky R, Chua KF, Lombard DB, et al. Genomic instability and aging-like phenotype in the absence of mammalian SIRT6. Cell 2006; 124:315–329. 16439206 10.1016/j.cell.2005.11.044

[bib21] 21Mao ZY, Hine C, Tian X, et al. SIRT6 promotes DNA repair under stress by activating PARP1. Science 2011; 332:1443–1446. 21680843 10.1126/science.1202723 PMC5472447

[bib22] 22Van Meter M, Kashyap M, Rezazadeh S, et al. SIRT6 represses LINE1 retrotransposons by ribosylating KAP1 but this repression fails with stress and age. Nat Commun 2014; 5: 5011. 25247314 10.1038/ncomms6011 PMC4185372

[bib23] 23Kawahara TLA, Michishita E, Adler AS, et al. SIRT6 links histone H3 lysine 9 deacetylation to NF-kappa B-dependent gene expression and organismal life span. Cell 2009; 136:62–74. 19135889 10.1016/j.cell.2008.10.052 PMC2757125

[bib24] 24Zhong L, D'Urso A, Toiber D, et al. The histone deacetylase Sirt6 regulates glucose homeostasis via Hif1 alpha. Cell 2010; 140:280–293. 20141841 10.1016/j.cell.2009.12.041 PMC2821045

[bib25] 25Sundaresan NR, Vasudevan P, Zhong L, et al. The sirtuin SIRT6 blocks IGF-Akt signaling and development of cardiac hypertrophy by targeting c-Jun. Nat Med 2012; 18:1643. 23086477 10.1038/nm.2961 PMC4401084

[bib26] 26Michishita E, McCord RA, Berber E, et al. SIRT6 is a histone H3 lysine 9 deacetylase that modulates telomeric chromatin. Nature 2008; 452:492–496. 18337721 10.1038/nature06736 PMC2646112

[bib27] 27Yang B, Zwaans BM, Eckersdorff M, Lombard DB. The sirtuin SIRT6 deacetylates H3 K56Ac *in vivo* to promote genomic stability. Cell cycle 2009; 8:2662–2663. 19597350 10.4161/cc.8.16.9329 PMC2728171

[bib28] 28Toiber D, Erdel F, Bouazoune K, et al. SIRT6 recruits SNF2H to DNA break sites, preventing genomic instability through chromatin remodeling. Mol Cell 2013; 51:454–468. 23911928 10.1016/j.molcel.2013.06.018 PMC3761390

[bib29] 29Michishita E, McCord RA, Boxer LD, et al. Cell cycle-dependent deacetylation of telomeric histone H3 lysine K56 by human SIRT6. Cell cycle 2009; 8:2664–2666. 19625767 10.4161/cc.8.16.9367 PMC4474138

[bib30] 30Hockemeyer D, Wang HY, Kiani S, et al. Genetic engineering of human pluripotent cells using TALE nucleases. Nat Biotechnol 2011; 29:731–734. 21738127 10.1038/nbt.1927 PMC3152587

[bib31] 31Liu GH, Suzuki K, Li M, et al. Modelling Fanconi anemia pathogenesis and therapeutics using integration-free patient-derived iPSCs. Nat Commun 2014; 5:4330. 24999918 10.1038/ncomms5330 PMC4291073

[bib32] 32Pan H, Zhang W, Zhang W, Liu GH. Find and replace: editing human genome in pluripotent stem cells. Protein Cell 2011; 2:950–956. 22173708 10.1007/s13238-011-1132-0 PMC4875250

[bib33] 33Nagai K, Matsushita T, Matsuzaki T, et al. Depletion of SIRT6 causes cellular senescence, DNA damage, and telomere dysfunction in human chondrocytes. Osteoarthritis Cartilage 2015; 23:1412–1420. 25819580 10.1016/j.joca.2015.03.024

[bib34] 34Wang D, Zhou J, Liu X, et al. Methylation of SUV39H1 by SET7/9 results in heterochromatin relaxation and genome instability. Proc Natl Acad Sci USA 2013; 110:5516–5521. 23509280 10.1073/pnas.1216596110 PMC3619320

[bib35] 35You BR, Shin HR, Han BR, Park WH. PX-12 induces apoptosis in Calu-6 cells in an oxidative stress-dependent manner. Tumour Biol 2015; 36:2087–2095. 25391429 10.1007/s13277-014-2816-x

[bib36] 36You BR, Shin HR, Park WH. PX-12 inhibits the growth of A549 lung cancer cells via G2/M phase arrest and ROS-dependent apoptosis. Int J Oncol 2014; 44:301–308. 24172913 10.3892/ijo.2013.2152

[bib37] 37Kunkel MW, Kirkpatrick DL, Johnson JI, Powis G. Cell line-directed screening assay for inhibitors of thioredoxin reductase signaling as potential anti-cancer drugs. Anticancer Drug Des 1997; 12:659–670. 9448705

[bib38] 38Shibata M, Hakuno F, Yamanaka D, et al. Paraquat-induced oxidative stress represses phosphatidylinositol 3-kinase activities leading to impaired glucose uptake in 3T3-L1 adipocytes. J Biol Chem 2010; 285:20915–20925. 20430890 10.1074/jbc.M110.126482 PMC2898352

[bib39] 39Castello PR, Drechsel DA, Patel M. Mitochondria are a major source of paraquat-induced reactive oxygen species production in the brain. J Biol Chem 2007; 282:14186–14193. 17389593 10.1074/jbc.M700827200 PMC3088512

[bib40] 40Malhotra D, Portales-Casamar E, Singh A, et al. Global mapping of binding sites for Nrf2 identifies novel targets in cell survival response through ChIP-Seq profiling and network analysis. Nucleic Acids Res 2010; 38:5718–5734. 20460467 10.1093/nar/gkq212 PMC2943601

[bib41] 41Sebastian C, Zwaans BMM, Silberman DM, et al. The histone deacetylase SIRT6 is a tumor suppressor that controls cancer metabolism. Cell 2012; 151:1185–1199. 23217706 10.1016/j.cell.2012.10.047 PMC3526953

[bib42] 42Suttner DM, Dennery PA. Reversal of HO-1 related cytoprotection with increased expression is due to reactive iron. FASEB J 1999; 13:1800–1809. 10506583 10.1096/fasebj.13.13.1800

[bib43] 43Parfenova H, Basuroy S, Bhattacharya S, et al. Glutamate induces oxidative stress and apoptosis in cerebral vascular endothelial cells: contributions of HO-1 and HO-2 to cytoprotection. Am J Physiol Cell Physiol 2006; 290:C1399–C1410. 16371440 10.1152/ajpcell.00386.2005

[bib44] 44Turkseven S, Kruger A, Mingone CJ, et al. Antioxidant mechanism of heme oxygenase-1 involves an increase in superoxide dismutase and catalase in experimental diabetes. Am J Physiol Heart Circ Physiol 2005; 289:H701–H707. 15821039 10.1152/ajpheart.00024.2005

[bib45] 45Schwer B, Schumacher B, Lombard DB, et al. Neural sirtuin 6 (Sirt6) ablation attenuates somatic growth and causes obesity. Proc Natl Acad Sci USA 2010; 107:21790–21794. 21098266 10.1073/pnas.1016306107 PMC3003110

[bib46] 46Li Q, Zhou H, Wurtele H, et al. Acetylation of histone H3 lysine 56 regulates replication-coupled nucleosome assembly. Cell 2008; 134:244–255. 18662540 10.1016/j.cell.2008.06.018 PMC2597342

[bib47] 47Ram O, Goren A, Amit I, et al. Combinatorial patterning of chromatin regulators uncovered by genome-wide location analysis in human cells. Cell 2011; 147:1628–1639. 22196736 10.1016/j.cell.2011.09.057 PMC3312319

[bib48] 48Liu GH, Suzuki K, Qu J, et al. Targeted gene correction of laminopathy-associated LMNA mutations in patient-specific iPSCs. Cell Stem Cell 2011; 8:688–694. 21596650 10.1016/j.stem.2011.04.019 PMC3480729

[bib49] 49Patsch C, Challet-Meylan L, Thoma EC, et al. Generation of vascular endothelial and smooth muscle cells from human pluripotent stem cells. Nat Cell Biol 2015; 17:994–U294. 26214132 10.1038/ncb3205 PMC4566857

[bib50] 50Debacq-Chainiaux F, Erusalimsky JD, Campisi J, Toussaint O. Protocols to detect senescence-associated beta-galactosidase (SA-betagal) activity, a biomarker of senescent cells in culture and *in vivo*. Nat Protoc 2009; 4:1798–1806. 20010931 10.1038/nprot.2009.191

[bib51] 51Kawata K, Kobayashi Y, Souda K, et al. Enhanced hepatic Nrf2 activation after ursodeoxycholic acid treatment in patients with primary biliary cirrhosis. Antioxid Redox Signal 2010; 13:259–268. 20055754 10.1089/ars.2009.2903

[bib52] 52Cullinan SB, Gordan JD, Jin JO, Harper JW, Diehl JA. The Keap1-BTB protein is an adaptor that bridges Nrf2 to a Cul3-based E3 ligase: Oxidative stress sensing by a Cul3-Keap1 ligase. Mol Cell Biol 2004; 24:8477–8486. 15367669 10.1128/MCB.24.19.8477-8486.2004 PMC516753

[bib53] 53Shi XB, Hong T, Walter KL, et al. ING2 PHD domain links histone H3 lysine 4 methylation to active gene repression. Nature 2006; 442:96–99. 16728974 10.1038/nature04835 PMC3089773

[bib54] 54Dahl JA, Collas P. A rapid micro chromatin immunoprecipitation assay (mu ChIP). Nat Protoc 2008; 3:1032–1045. 18536650 10.1038/nprot.2008.68

[bib55] 55Zhang Y, Liu T, Meyer CA, et al. Model-based analysis of ChIP-Seq (MACS). Genome Biol 2008; 9:R137. 18798982 10.1186/gb-2008-9-9-r137 PMC2592715

[bib56] 56Trapnell C, Pachter L, Salzberg SL. TopHat: discovering splice junctions with RNA-Seq. Bioinformatics 2009; 25:1105–1111. 19289445 10.1093/bioinformatics/btp120 PMC2672628

[bib57] 57Roberts A, Pimentel H, Trapnell C, Pachter L. Identification of novel transcripts in annotated genomes using RNA-Seq. Bioinformatics 2011; 27:2325–2329. 21697122 10.1093/bioinformatics/btr355

[bib58] 58Trapnell C, Roberts A, Goff L, et al. Differential gene and transcript expression analysis of RNA-seq experiments with TopHat and Cufflinks. Nat Protoc 2012; 7:562–578. 22383036 10.1038/nprot.2012.016 PMC3334321

[bib59] 59Li H, Durbin R. Fast and accurate long-read alignment with Burrows-Wheeler transform. Bioinformatics 2010; 26:589–595. 20080505 10.1093/bioinformatics/btp698 PMC2828108

