# Supplementary material for: SIRT6 safeguards human mesenchymal stem cells from oxidative stress by coactivating NRF2
Source: Cell Res. 2016 Jan 15;26(2):190–205. doi: 10.1038/cr.2016.4 (PMC4746611; doi:10.1038/cr.2016.4)
Supplement: Supplementary information, Figure S1 — Characterization of SIRT6-deficient hESCs and differentiation of hESCs to hMSCs. [file cr20164x1.pdf]

Supplementary information, Figure S1

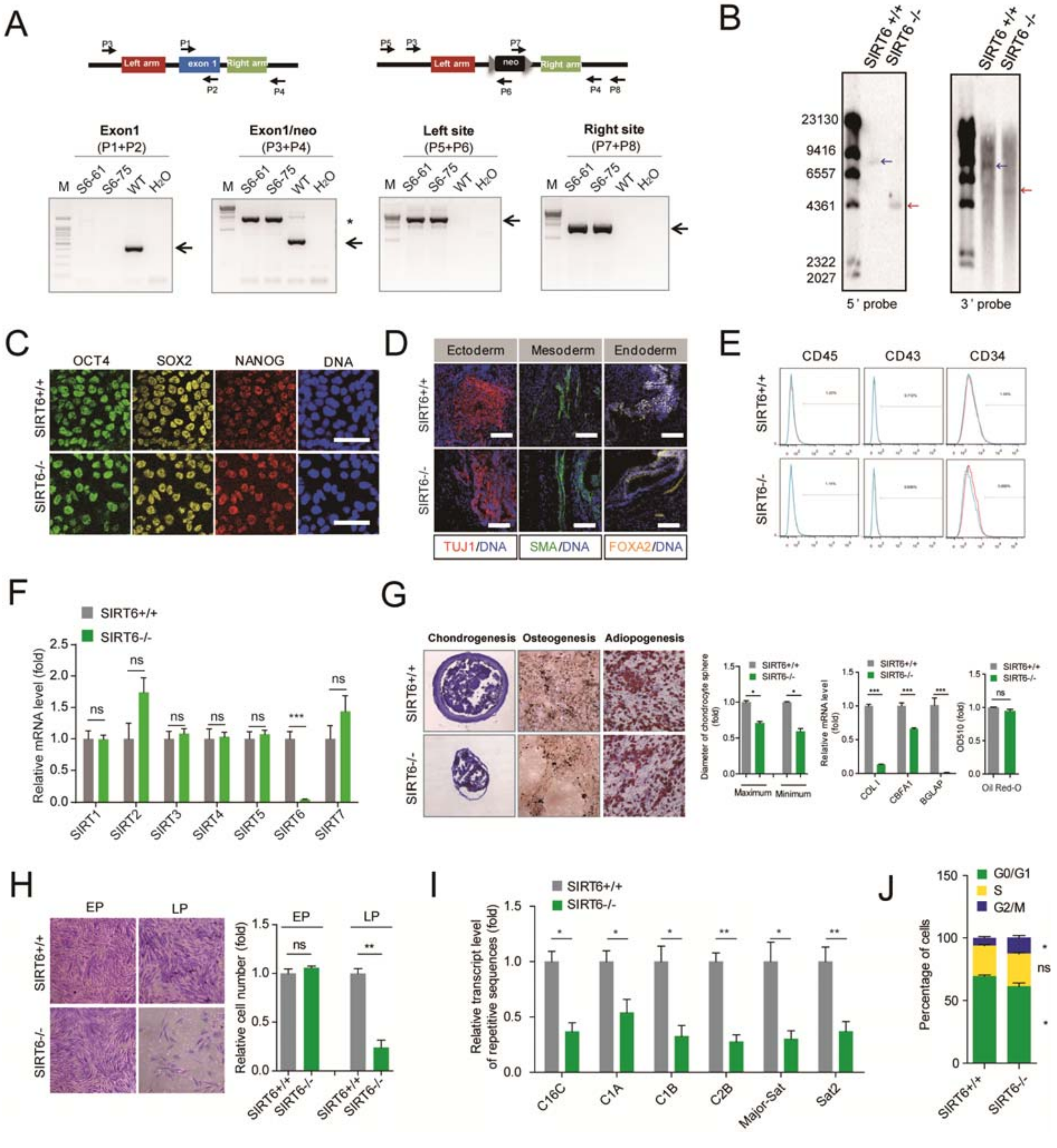

Supplementary information, Figure S1 Characterization of SIRT6-deficient hESCs and differentiation of hESCs to hMSCs.

(A) Genomic PCR analyses of WT and SIRT6-deficient hESCs using primer pairs as following. Primer pair (P1+P2) were used to amplify exon 1 of *SIRT6* (arrow) (panel 1); Primer pair (P3+P4) were used to amplify exon 1 of *SIRT6* (arrow) and neo (star), and the neo band indicated that the exon 1 in both *SIRT6* alleles were replaced by neo (panel 2); Primer pair (P5+P6) were used to amplify 5' homology arm (arrow, panel 3); Primer pair (P7+P8) were used to amplify 3' homology arm (arrow, panel 4). Two different *SIRT6*-targeted clones (S6-61 and S6-75) were indicated. **(B)** Southern blot

analysis of WT and SIRT6-deficient hESCs was performed using 5' probe and 3' probe, respectively. Genomic DNA was digested by Hind III and Xho I to generate one fragment (about 7.7 kb) in WT hESCs (indicated by the blue arrow in the left and right panels), which was recognized by 5' probe at 5' site of the segment and 3' probe at 3' site of the segment, respectively. Genomic DNA from SIRT6-deficient hESCs was digested to two fragments by Hind III and Xho I. 5' fragment was around 3.8 kb and recognized by 5' probe (indicated by the red arrow in the left panel), and 3' fragment was around 5.1 kb and recognized by 3' probe (indicated by the red arrow in the right panel). Corresponding DNA markers (kb) were indicated. **(C)** Immunofluorescence analysis indicating the expression of pluripotency markers in WT and SIRT6-deficient hESCs. Scale bar, 20 μm. **(D)** Immunostaining images showing *in vivo* differentiation potential to ectodermal (TUJ1, red), mesodermal (SMA, green) and endodermal (FOXA2, yellow) tissues in teratomas derived from SIRT6-deficient hESCs. Scale bar, 100 μm. **(E)** FACS analysis showing the absence of CD45, CD43, and CD34 cell surface markers in WT and SIRT6-deficient hMSCs. **(F)** RT-qPCR analysis indicating specific downregulation of SIRT6 in SIRT6-deficient hMSCs. Data were presented as mean ± SEM, n=3, ns, not significant, ***p<0.001. **(G)** Capabilities of WT and SIRT6-deficient hMSCs in chondrogenesis, osteogenesis, and adiopogenesis, respectively. Alcian blue, Von Kossa, and Oil Red-O were used to characterize chondrocytes, osteoblasts, and adipocytes, respectively. Measurement of the diameters of chondrocyte spheres indicated an abnormal chondrogenesis from SIRT6-deficient hMSCs. RT-qPCR analyses showed impaired expression of osteoblast-specific markers COL1A1 (COL1), CBFA-1, and BGLAP in the osteoblast derivatives differentiated from SIRT6-deficient hMSCs. The OD510 values of Oil Red-O staining in WT and SIRT6-deficient adipocytes were comparable. Data were presented as mean ± SEM, n=3, ns, not significant, *p<0.05, ***p<0.001. **(H)** Cell proliferation analysis of WT and SIRT6-deficient hMSCs performed at early (EP, passage 6) and late passages (LP, passage 9), respectively. Cells were stained with crystal violet after a two-week culture and the numbers of the crystal violet-positive cells were quantified. Data were presented as mean ± SEM, n=3, ns, not significant, **p<0.01. **(I)** RT-qPCR analysis of the transcripts from centromeric repetitive sequences. C: centromere; Sat: satellite sequences. Values were normalized against GAPDH. Data were presented as mean ± SEM, n=3, *p<0.05, **p<0.01. **(J)** Cell cycle profiles of WT and SIRT6-deficient hMSCs. Data were presented as mean ± SEM, n=3, ns, not significant, *p<0.05.
