# Supplementary material for: SIRT6 safeguards human mesenchymal stem cells from oxidative stress by coactivating NRF2
Source: Cell Res. 2016 Jan 15;26(2):190–205. doi: 10.1038/cr.2016.4 (PMC4746611; doi:10.1038/cr.2016.4)
Supplement: Supplementary information, Figure S2 — Characterization of SIRT6-deficient hMSCs. [file cr20164x2.pdf]

Supplementary information, Figure S2

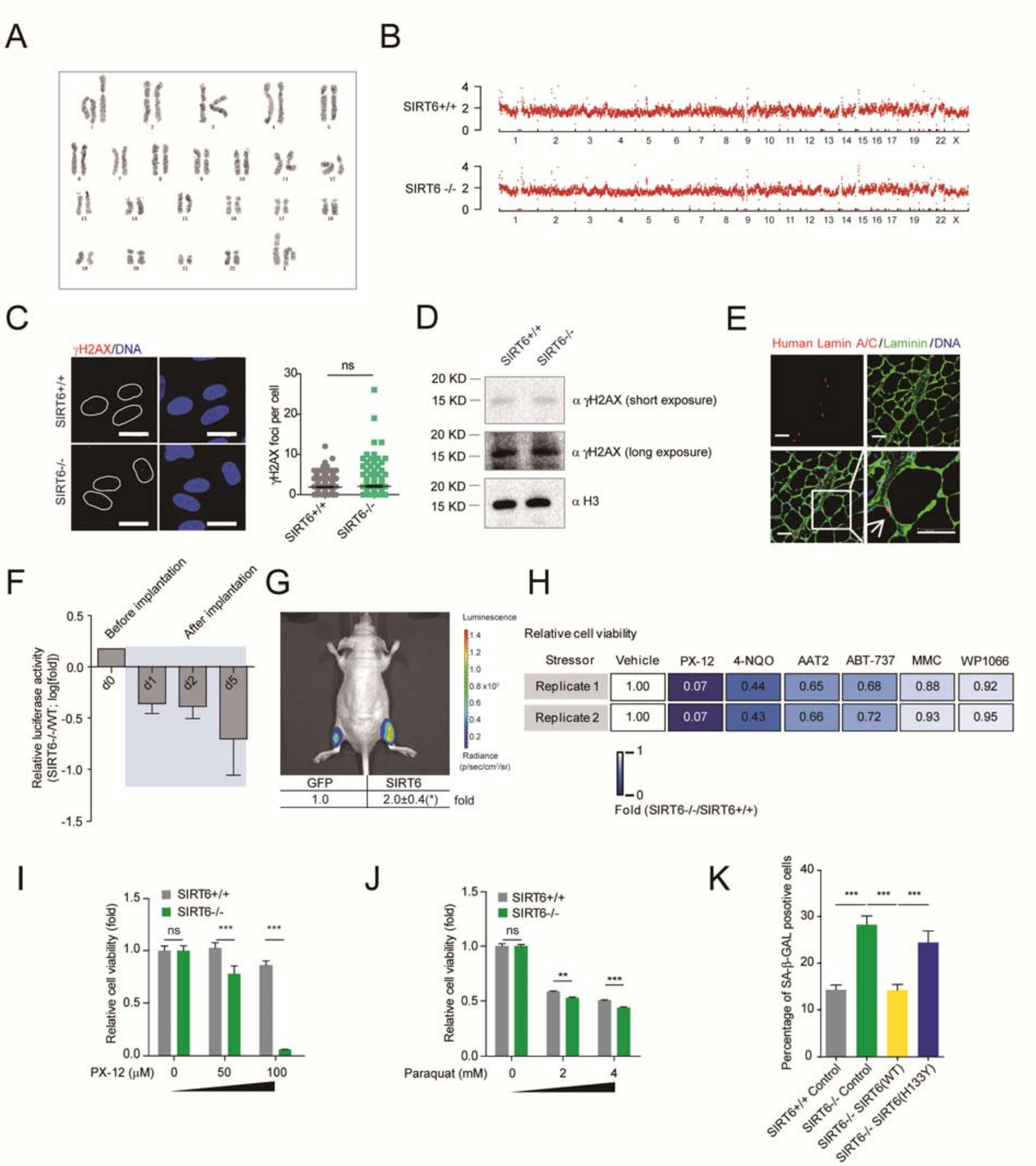

Supplementary information, Figure S2 Characterization of SIRT6-deficient hMSCs.

(A) Karyotyping analysis of SIRT6-deficient hMSCs indicating normal karyotype. Within the population of SIRT6-deficient hMSCs with the normal chromosome number which were used for detailed analysis, 35/35 cells (100%) showed normal chromosomal morphology. **(B)** Genome-wide copy number variations (CNVs) analysis of the SIRT6-deficient and WT hMSCs. No significant CNV alterations between SIRT6-deficient and WT hMSCs were found. **(C)** Immunofluorescence analysis of γH2AX in WT and SIRT6-deficient hMSCs (left) and statistical analysis of the γH2AX foci in the

nuclei (right). The cell number used for analysis was 168, and the median values of both WT and SIRT6-deficient hMSCs were 2. Scale bar, 20 μm. **(D)** Western blotting analysis showing comparable γH2AX levels in WT and SIRT6-deficient hMSCs. Histone 3 (H3) was used as the loading control. **(E)** Immunofluorescence images showing integration of human nuclei (arrow) into WT hMSC-implanted mouse TA muscle. Human Lamin A/C (red) indicated integrated human cells, and laminin (green) indicated mouse muscle tissue. Scale bar, 50 μm. **(F)** Analysis of luciferase activity in mouse TA muscles by IVIS showing premature attrition of SIRT6-deficient hMSCs in an *in vivo* niche (also see Figure 1I). Luciferase activities were determined before implantation (d0), as well as 1 day (d1), 2 days (d2), and 5 days (d5) after implantation. Data were presented as the ratios of SIRT6-/- to WT (log [fold]), mean ± SD, n=4. **(G)** Measurement of luciferase activity in mouse TA muscles by IVIS. 2×10^6 of SIRT6-deficient hMSCs overexpressing GFP plus luciferase (control group, left) and SIRT6-deficient hMSCs overexpressing SIRT6 plus luciferase (right) were implanted into the TA muscles of mice, respectively. 1 week after implantation, mice were intraperitoneally injected with PX-12 (20 mg/kg) for 24 hours, and then luciferase activities were measured. Data were presented as mean ± SEM, n=4, *p<0.05. **(H)** Cell viability of WT and SIRT6-deficient hMSCs under various stress conditions was determined by MTS assay. The values obtained from vehicle (DMSO)-treated group were normalized to 1. Data from two biological replicates were presented. **(I)** and **(J)** WT and SIRT6-deficient hMSCs were treated with the indicated concentrations of PX-12 (I) or paraquat (J) for 24 hours, and then the relative cell viability was measured by MTS assay. Cell viability without PX-12 or paraquat treatment was normalized to 1. Data were presented as mean ± SEM, n=5, ns, not significant, **p<0.01, ***p<0.001. **(K)** Overexpression of SIRT6 (WT), not SIRT6 (HY), in SIRT6-deficient hMSCs at passage 9 repressed accelerated cellular senescence. SA-β-Gal activity was analyzed at passage 10. Data were presented as mean ± SEM, n=5, ***p<0.001.
