# Supplementary material for: SIRT6 safeguards human mesenchymal stem cells from oxidative stress by coactivating NRF2
Source: Cell Res. 2016 Jan 15;26(2):190–205. doi: 10.1038/cr.2016.4 (PMC4746611; doi:10.1038/cr.2016.4)
Supplement: Supplementary information, Figure S3 — Gene expression analyses of WT and SIRT6-deficient hMSCs. [file cr20164x3.pdf]

Supplementary information, Figure S3

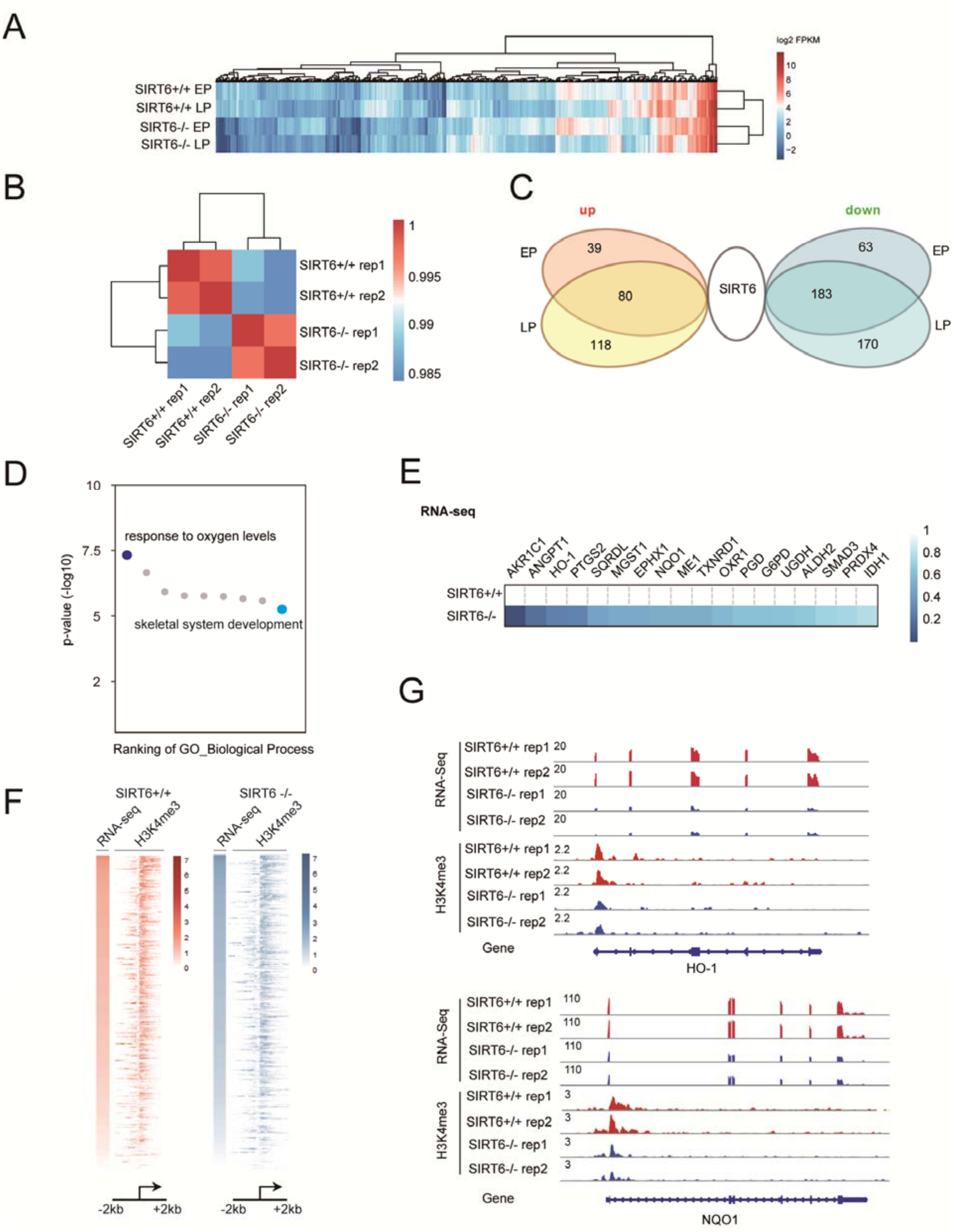

Supplementary information, Figure S3 Gene expression analyses of WT and SIRT6-deficient hMSCs.

(A) Heatmap showing the expression profiles of significantly up- and down-regulated genes in SIRT6-deficient hMSCs comparing to WT hMSCs in the early passage (passage 6), or significantly up- and down-regulated genes in the late passage (passage 9) hMSCs comparing to the early passage counterparts. **(B)** Heatmap showing high correlation between the biological replicates. **(C)** Venn diagram showed that the early passage (EP) and late passage (LP) hMSCs shared significantly up- or down-regulated genes in SIRT6-deficient hMSCs relative to WT hMSCs. **(D)** GO analysis of the significantly down-regulated genes in hMSCs upon SIRT6 depletion. **(E)** Heatmap showing fold change between SIRT6-deficient and WT hMSCs indicated the decreased expression of NRF2 target genes in SIRT6-deficient hMSCs. **(F)** Heatmap showing the H3K4me3 levels at the gene promoters ordered by the expression of the corresponding genes. **(G)** Examples of two representative NRF2 target genes showing diminished gene expression and decreased H3K4me3 modification at promoter regions.
