# Supplementary material for: SIRT6 safeguards human mesenchymal stem cells from oxidative stress by coactivating NRF2
Source: Cell Res. 2016 Jan 15;26(2):190–205. doi: 10.1038/cr.2016.4 (PMC4746611; doi:10.1038/cr.2016.4)
Supplement: Supplementary information, Figure S4 — Gene expression and epigenome analyses of WT and SIRT6-deficient hMSCs. [file cr20164x4.pdf]

Supplementary information, Figure S4

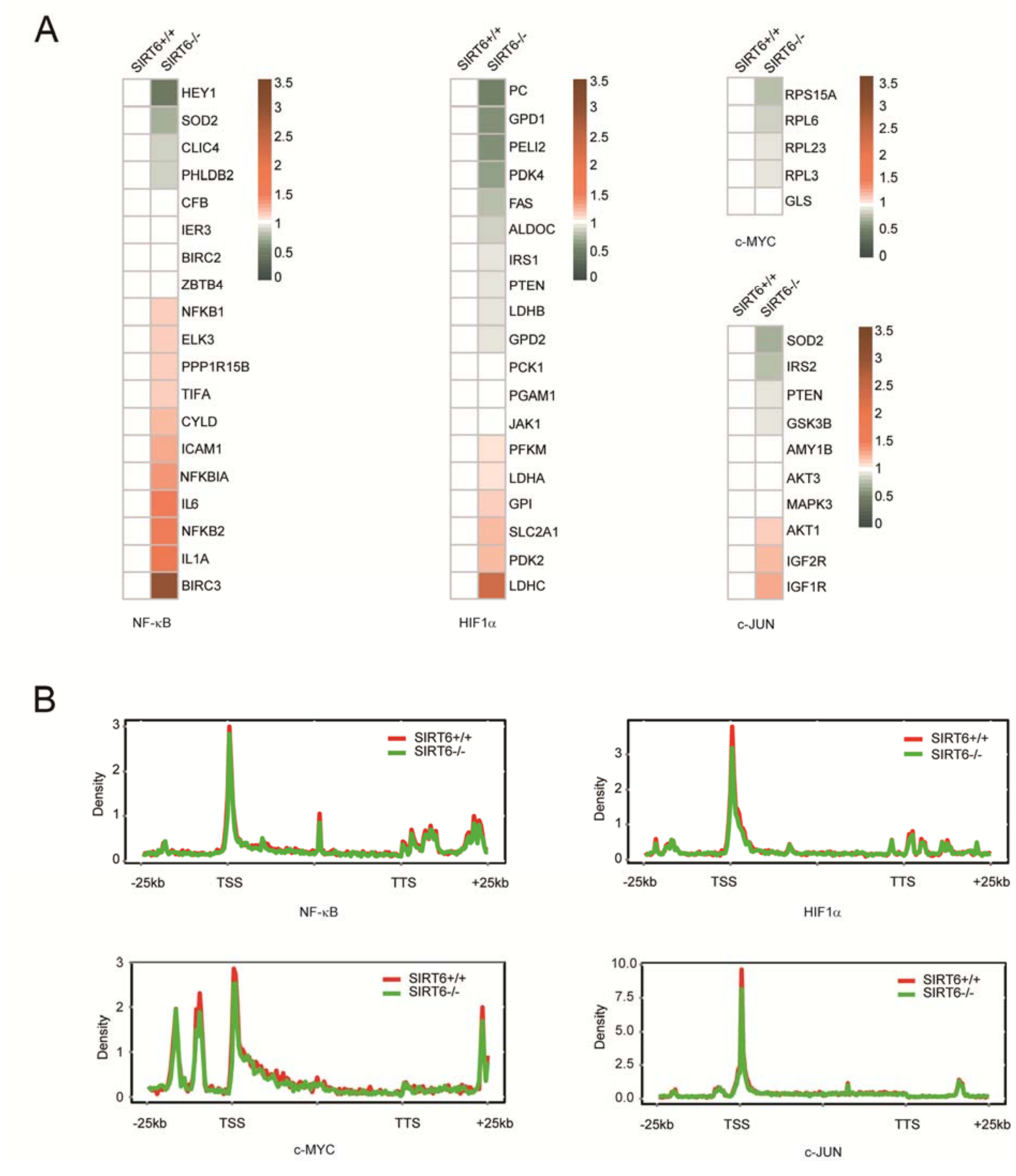

Supplementary information, Figure S4 Gene expression and epigenome analyses of WT and SIRT6-deficient hMSCs.

(A) Heatmap showing fold changes of NF-κB, HIF1α, c-MYC, and c-JUN's target genes between SIRT6-deficient and WT hMSCs. **(B)** Average profiles of the H3K4me3 histone modification around the NF-κB, HIF1α, c-MYC, and c-JUN's target gene body regions in SIRT6-deficient and WT hMSCs. TSS, transcription start site; TTS, transcription termination site.
