# Supplementary material for: SIRT6 safeguards human mesenchymal stem cells from oxidative stress by coactivating NRF2
Source: Cell Res. 2016 Jan 15;26(2):190–205. doi: 10.1038/cr.2016.4 (PMC4746611; doi:10.1038/cr.2016.4)
Supplement: Supplementary information, Figure S5 — SIRT6 regulates recruitment of RNAP II to HO-1 promoter in a deacetylase-dependent manner. [file cr20164x5.pdf]

Supplementary information, Figure S5

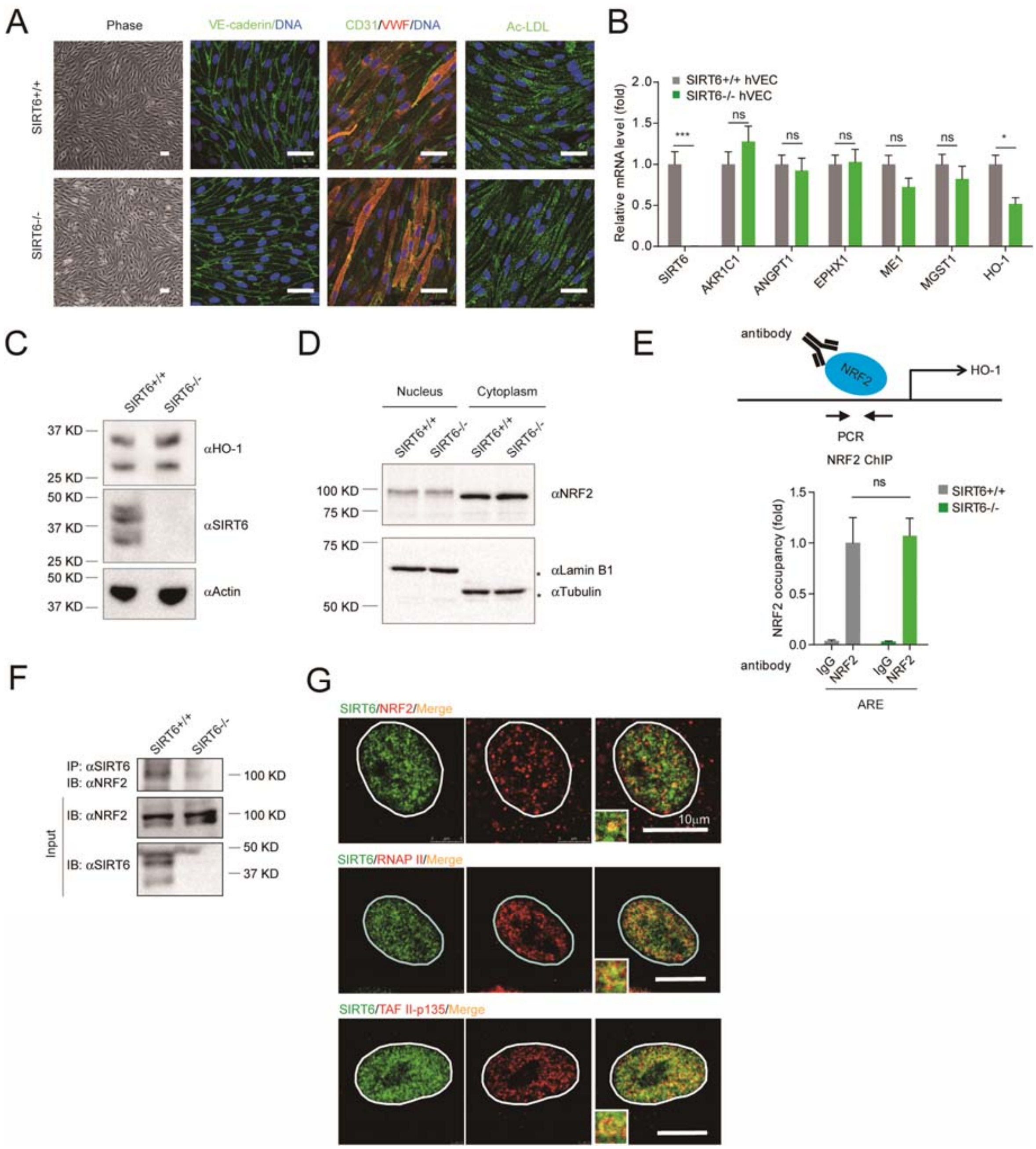

Supplementary information, Figure S5 SIRT6 regulates recruitment of RNAP II to HO-1 promoter in a deacetylase-dependent manner.

(A) Bright field and VE-caderin, CD31, and VWF immunofluorescence micrographs of the human vascular endothelial cells (hVECs) derived from WT and SIRT6-deficient hESCs. The functionality of hVECs was confirmed by uptake of acetylated low density lipoproteins (Ac-LDL). DNA was visualized by Hoechst 33342. Bright field scale bar, 100 μm; fluorescence scale bar, 50 μm. **(B)** RT-qPCR analysis of NRF2 target genes in WT and SIRT6-deficient hVECs. Values were normalized against 18S rRNA. Data were presented as mean ± SEM, n=3, ns, not significant, *p<0.05,

***p<0.001. **(C)** Western blotting analysis of HO-1 showing comparable levels between WT and SIRT6-deficient hVECs. β-Actin was used as the loading control. **(D)** Western blotting analysis of nuclear and cytosolic NRF2 levels in WT and SIRT6-deficient hMSCs. β-tubulin and Lamin B1 were used as loading controls for cytosolic and nuclear extracts, respectively. **(E)** ChIP-qPCR assay indicating comparable NRF2 occupancy at the ARE of the HO-1 promoter between WT and SIRT6-deficient hMSCs. Data were presented as mean ± SEM, n=3, ns, not significant. **(F)** Co-IP showing the presence of NRF2 in anti-SIRT6 immunoprecipitates in WT hMSCs. **(G)** Immunofluorescence analysis showing partial colocalization (yellow) of SIRT6 (green) with NRF2 (red), RNAP II (red), or TAF II-p135 (red) in the WT hMSCs. Scale bar, 10 μm.
