# Supplementary material for: SIRT6 safeguards human mesenchymal stem cells from oxidative stress by coactivating NRF2
Source: Cell Res. 2016 Jan 15;26(2):190–205. doi: 10.1038/cr.2016.4 (PMC4746611; doi:10.1038/cr.2016.4)
Supplement: Supplementary information, Figure S6 — HO-1 overexpression rescues premature attrition of SIRT6-deficient hMSCs in vivo. [file cr20164x6.pdf]

Supplementary information, Figure S6

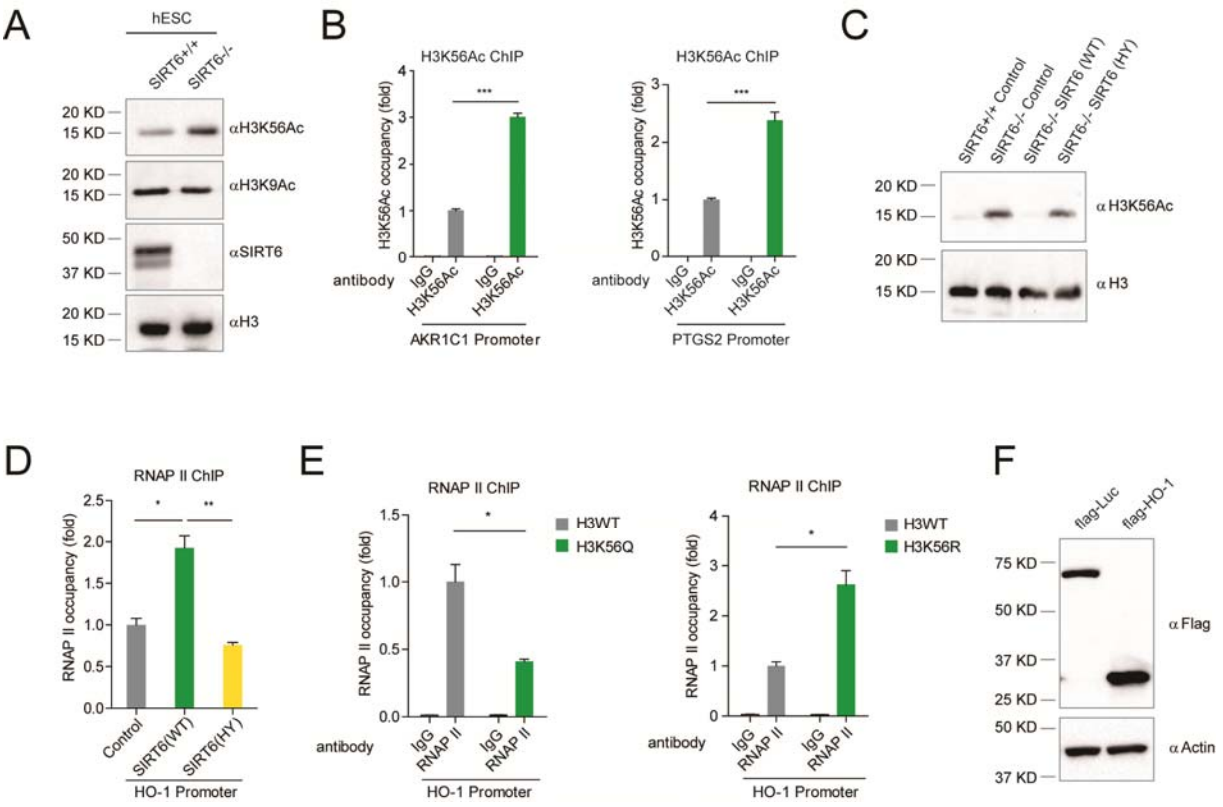

Supplementary information, Figure S6 HO-1 overexpression rescues premature attrition of SIRT6-deficient hMSCs *in vivo*.

(A) Western blots showing upregulation of H3K56Ac, rather than H3K9Ac in SIRT6-deficient hESCs relative to their WT counterparts. Histone 3 (H3) was used as the loading control. **(B)** ChIP-qPCR analysis showing an increase in the enrichment of acetylated H3K56 at both AKR1C1 and PTGS2 promoters in SIRT6-deficient hMSCs. Data were presented as mean ± SEM, n=3, ***$p<0.001$. **(C)** Western blotting analysis of H3K56Ac in the protein extracts from WT and SIRT6-deficient hMSCs overexpressing luciferase (Control), SIRT6 (WT), and SIRT6 (HY). Histone 3 (H3) was used as the loading control. **(D)** ChIP-qPCR analysis of RNAP II enrichment at HO-1 promoter in SIRT6-deficient hMSCs transduced with lentiviral vector encoding luciferase (Control), SIRT6 (WT), and SIRT6 (HY). Data were presented as mean ± SEM, n=3, *$p<0.05$, **$p<0.01$. **(E)** Left panel: ChIP-qPCR analysis of RNAP II enrichment at HO-1 promoter in WT hMSCs transduced with lentiviral vector encoding H3WT or H3K56Q. Right panel: ChIP-qPCR analysis of RNAP II enrichment at HO-1 promoter in SIRT6-deficient hMSCs transduced with lentiviral vector encoding H3WT or H3K56R. Data were presented as mean ± SEM, n=3, *$p<0.05$. **(F)** Western blotting analysis of the protein extracts from SIRT6-deficient hMSCs transduced with flag-luciferase (flag-Luc) or flag-HO-1 with an anti-flag antibody. β-Actin was used as the loading control.
